# FilTar: using RNA-Seq data to improve microRNA target prediction accuracy in animals

**DOI:** 10.1093/bioinformatics/btaa007

**Published:** 2020-01-13

**Authors:** Thomas Bradley, Simon Moxon

**Affiliations:** b1 School of Biological Sciences, University of East Anglia, Norwich NR4 7TJ, UK; b2 Earlham Institute, Norwich Research Park, Norwich NR4 7UZ, UK

## Abstract

**Motivation:**

MicroRNA (miRNA) target prediction algorithms do not generally consider biological context and therefore generic target prediction based on seed binding can lead to a high level of false-positive predictions. Here, we present FilTar, a method that incorporates RNA-Seq data to make miRNA target prediction specific to a given cell type or tissue of interest.

**Results:**

We demonstrate that FilTar can be used to: (i) provide sample specific 3′-UTR reannotation; extending or truncating default annotations based on RNA-Seq read evidence and (ii) filter putative miRNA target predictions by transcript expression level, thus removing putative interactions where the target transcript is not expressed in the tissue or cell line of interest. We test the method on a variety of miRNA transfection datasets and demonstrate increased accuracy versus generic miRNA target prediction methods.

**Availability and implementation:**

FilTar is freely available and can be downloaded from https://github.com/TBradley27/FilTar. The tool is implemented using the Python and R programming languages, and is supported on GNU/Linux operating systems.

**Supplementary information:**

Supplementary data are available at *Bioinformatics* online.

## 1 Introduction

MicroRNAs (miRNAs) exert widespread post-transcriptional control over mRNA expression in most animal lineages (Bartel, 2018), creating a need for the accurate identification of miRNA targets in order to better understand gene regulation. Traditional methods for providing experimental support for putative interactions include the use of reporter assays to test for a direct interaction between the miRNA and mRNA, or perturbation experiments to test for the effect of increased or decreased miRNA levels on target mRNA, or the corresponding proteins translated from these molecules (Kuhn *et al.*, 2008). More recent methods allow researchers to test for direct interactions between miRNA and putative targets using transcriptome-wide crosslinking and immunoprecipitation experiments. These methods usually test for binding between the putative miRNA target and argonaute (AGO) (Chi *et al.*, 2009; König *et al.*, 2010; Van Nostrand *et al.*, 2016), a key component of the miRNA-guided RISC (RNA-induced silencing complex), and in addition, some methods can be used to determine the identity of the miRNA which is guiding AGO to the target transcript (Helwak and Tollervey, 2014; Kudla *et al.*, 2011).

Currently available data for these types of experiments are generally limited in number and diversity of cell types and species. Inspection of the TarBase resource (v8.0) (Karagkouni *et al.*, 2018), a database of published, experimentally supported miRNA interactions, reveals that, at the time of writing, even for a widely utilized model organism such as mouse, AGO immunoprecipitation datasets are available for only three cell lines and five tissues. The problem is exacerbated when examining records for other model organisms such as rat and zebrafish, in which no data from immunoprecipitation experiments are reported. This is likely because generating data of this type is usually prohibitively expensive in terms of skills, time and material resources needed to complete sophisticated transcriptome-wide, next-generation library preparation and sequencing protocols. The limited applicability of experimental approaches, therefore, underlies the continuing necessity of computational approaches for predicting miRNA targets.

There are a number of existing computational tools for predicting miRNA targets in animals. Algorithms such as TargetScan use complementarity between the seed sequence of the miRNA (Bartel, 2018; Lewis *et al.*, 2003) and a corresponding region of the 3′-UTR of its target as the basis of target prediction (Agarwal *et al.*, 2015; Friedman *et al.*, 2008; Garcia *et al.*, 2011; Grimson *et al.*, 2007; Lewis *et al.*, 2003, 2005). Alternatively, some miRNA target prediction algorithms do not require full complementarity in the miRNA seed region (Enright *et al.*, 2003; Gumienny and Zavolan, 2015; John *et al.*, 2004; Khorshid *et al.*, 2013; Wang, 2016), or predict miRNA targeting to occur in the coding region of the transcript as well as the 3′-UTR (Reczko *et al.*, 2012). Most algorithms, in addition to considerations of seed complementarity, and the location of the target site within the transcript, also consider features such as the conservation of the miRNA target site in closely related species, the thermodynamic stability of the miRNA–mRNA duplex, and the structural accessibility of putative target sites to the miRNA–RISC complex, as variables which are also thought to influence miRNA targeting and subsequent transcript repression (Ritchie and Rasko, 2014).

Although intramolecular features are often considered, current miRNA target predictions currently do not account for the broader cellular context in which miRNA targeting occurs. The clearest indication of this is that current target prediction tools do not account for whether predicted targets are expressed within a given cell type or tissue. If the predicted target is not expressed, it cannot physically interact and be translationally inhibited or repressed by miRNA molecules. As expression profiles vary across different cell types and tissues, failing to consider whether a predicted target is expressed in a given cellular context may lead to false-positive results when making miRNA target predictions.

For the prediction of miRNA targets in the 3′-UTR, an additional complication is that the identity of an individual 3′-UTR may not be constant across different cell types or different biological conditions due to alternative cleavage and polyadenylation (APA) (Elkon *et al.*, 2013; Tian and Manley, 2017). APA is the process by which cellular polyadenylation machinery utilizes alternative polyadenlyation sites located on precursor mRNA molecules to produce transcripts with alternative 3′-UTR sequences. Differential usage of polyadenylation sites in diverse tissues or biological conditions, can result in distinct 3′-UTR isoform abundance profiles existing between different cell types (Nam *et al.*, 2014). One consequence of the existence of 3′-UTR isoforms is that a miRNA target site may exist for some 3′-UTR isoforms of the same annotated mRNA but not others.

As a result, APA allows the differential usage of miRNA target sites by the cell, diversifying and modifying the effect of miRNAs in different cellular contexts. For example, in cancer cells, shortening of 3′-UTRs can activate oncogenes by increasing mRNA stability, partially through the reduction in the number of miRNA target sites in their 3′-UTRs, decreasing the extent to which they are repressed (Mayr and Bartel, 2009). In contrast, an extensive enrichment of longer 3′-UTRs and hence additional miRNA target sites have been discovered in mammalian brain tissue (Miura *et al.*, 2013), which has been hypothesized to serve as an extended platform for the regulation of gene expression (Wang and Yi, 2014). This evidence of context-specific miRNA action underlies the utility of methods which accounts for this information in order to increase the precision and sensitivity of miRNA target predictions.

Most databases of miRNA target predictions do not incorporate information relating to APA, and instead rely on default 3′-UTR annotations provided by public sequence databases such as Ensembl (Birney, 2004; Cunningham *et al.*, 2019) and RefSeq (Pruitt *et al.*, 2007, 2014), when identifying potential miRNA targets. Similarly, most prediction algorithms do not easily allow the user to generate predictions for multiple 3′-UTR isoforms of the same mRNA. An exception is TargetScan (v7) (Agarwal *et al.*, 2015). In this version, each mRNA transcript is associated with a distinct profile of relative 3′-UTR isoform abundances. From this profile, each scored target site is weighted by the abundance of the 3′-UTR segment containing the predicted target site relative to all 3′-UTRs of that transcript. The caveat of this analysis being that 3′-UTR profiles are generated from sequencing data obtained from only four human cell lines (Nam *et al.*, 2014), which is subsequently treated as being representative for all cell types. Although it was shown that this approach was superior to not incorporating 3′-UTR profile data at all, it was sub-optimal in comparison to using 3′-UTR profiles specific to each cellular context examined (Nam *et al.*, 2014). Crucially, a miRNA target prediction tool which enables the user to predict miRNA targets specific to a given tissue or cell line is currently lacking.

Presented in this article is FilTar, a tool which takes RNA-Seq data as input and generates miRNA target predictions tailored to specific cellular contexts. Specificity of target prediction is increased by utilising information from sequencing data both to filter out poorly or non-expressed targets and to refine 3′-UTR annotations. Analysis demonstrates that predicted miRNA targets gained and lost due to 3′-UTR reannotation behave like pre-existing predicted miRNA target and non-targets, respectively, in response to miRNA transfection. The cumulative effect of integrating these additional processing steps into conventional miRNA target prediction workflows is to increase prediction accuracy and to drastically alter the number of miRNA target predictions made between different cell types.

## 2 Materials and methods

All following steps were carried out using the FilTar tool. The workflow and parameters are described in detail below.

### 2.1 Implementation

FilTar is a command line tool for GNU/Linux operating systems written predominantly in the Python (v3.6.8) and R (v3.5.0) programming languages. Users can configure the tool to process available RNA-Seq datasets from public repositories such as the European Nucleotide Archive (ENA; https://www.ebi.ac.uk/ena) (Harrison *et al.*, 2019; Leinonen *et al.*, 2011) and the Sequence Read Archive (SRA; https://ncbi.nlm.nih.gov/sra) (Leinonen *et al.*, 2010), and also the user’s own private sequencing data. All reported parameters are fully configurable within the FilTar tool. FilTar utilizes Snakemake (v5.4.0) (Köster and Rahmann, 2012) for workflow management. Most FilTar dependencies are managed using Conda (v4.6.6; https://docs.conda.io/en/latest/).

### 2.2 Data preprocessing

Reads were trimmed using Trim Galore (v0.5.0) (Krueger, 2015), a wrapper around Cutadapt (v1.16) (Martin, 2011), using default parameters with the exception of the ‘length’ and ‘stringency’ parameters which were set to 35 and 4, respectively.

### 2.3 3′-UTR reannotation

In order to build an index for the alignment of FASTQ reads to the genome, unmasked chromosomal reference genome assembly fasta files for human (GRCh38.p12) and mouse (GRCm38.p6) (Schneider *et al.*, 2017) were downloaded from release 94 of Ensembl (www.ensembl.org/index.html) (Cunningham *et al.*, 2019). All subsequent files obtained from the Ensembl resource were for this same release version. Splice-aware mapping of reads to the genome was achieved using HISAT2 (v2.1.0) (Kim *et al.*, 2015): The locations of exons and junction sites were determined by running the appropriate HISAT2 scripts on the relevant species-specific GTF (gene transfer format) annotation file also obtained from Ensembl. The ‘hisat2-build’ binary was executed using the ‘ss’ and ‘exon’ flags indicating splice site and exon co-ordinates built from the previous step.

The indexed genome was used for FASTQ read alignment using the ‘hisat2’ command. The ‘rna-strandness’ option was used for strand-aware alignment. The strandedness of RNA-seq datasets was determined using the ‘quant’ command of the Salmon (v0.11.3) (Patro *et al.*, 2017) RNA-seq quantification tool, by setting the ‘lib-type’ option to ‘A’ for automatic inference of library type. The SAMtools (v1.8) (Li *et al.*, 2010) ‘view’ and ‘sort’ commands were used to sort data from sam to bam format, and to sort the resultant bam files, respectively.

Sorted bam files were converted to bedgraph format using the ‘genomeCoverageBed’ command of bedtools (v2.27.1) (Quinlan, 2014; Quinlan and Hall, 2010) using the ‘bg’, ‘ibam’ and ‘split’ options. Bedgraph files representing biological replicates of the same condition were merged using bedtool’s ‘unionbedg’ command. FilTar then calculated the mean average coverage value for each record in the merged bedgraph file.

Existing transcript models were produced by converting Ensembl GTF annotations files (containing one or zero 3′-UTR annotations per protein-coding transcript) into genePred format using the UCSC ‘gtfToGenePred’ binary, and then from genePred format to bed12 format using the UCSC ‘genePredToBed’ binary (Kent *et al.*, 2002). APAtrap (Ye *et al.*, 2018), the 3′-UTR reannotation tool, was used to refine 3′-UTR annotations on a transcript-by-transcript basis by integrating information from the bed12 file and bedgraph files using the ‘identifyDistal3UTR.pl’ perl script with default parameters. FilTar then integrated existing transcript 3′-UTR models with the new models predicted by APAtrap—replacing existing 3′-UTR models for those transcripts in which APAtrap has made a reannotation. Only truncations or elongations of single exon 3′-UTR annotations were integrated into final 3′-UTR annotations; novel 3′-UTR predictions (*i.e.* prediction of 3′-UTRs for transcripts without a previous 3′-UTR annotation) were discarded and alterations of the 3′-UTR start site were also not permitted, due to the reannotation of 3′-UTR start sites by the APAtrap dependency as beginning at the start position of the final exon in standard Ensembl transcript models. No alterations to existing 3′-UTR annotations spanning multiple exons were permitted, as this is not intended functionality of the APAtrap tool.

### 2.4 miRNA target prediction

Target prediction for the analyses presented in this study was conducted using the TargetScan algorithm (v.7.01) (Agarwal *et al.*, 2015). Mature miRNA sequences were obtained from release 22 of miRBase (www.mirbase.org) (Griffiths‐Jones, 2004; Kozomara *et al.*, 2019). The 3′-UTR sequence data required for target prediction can either be provided as multiple sequence alignments (MSAs) or single sequences, with the former option enabling the computation of 3′-UTR branch lengths and the probability of conserved targeting (Pct) for putative miRNA target sites.

Multiple sequence alignments are derived from 100-way (human reference) and 60-way (mouse reference) whole-genome alignments hosted at the UCSC genome browser (https://genome.ucsc.edu) (Kent *et al.*, 2002) generated using the threaded blockset-aligner (Blanchette, 2004) stored in MAF (multiple alignment format) format. MAF files are indexed, and the relevant alignment regions corresponding to 3′-UTR co-ordinates extracted using ‘MafIO’ functions contained within the Biopython (v1.72) library (Cock *et al.*, 2009). For human MSAs, during post-processing, distantly related species were removed, resulting in 84-way MSAs (Agarwal *et al.*, 2015).

If MSAs are not used, single sequences are extracted from DNA files using relevant 3′-UTR co-ordinates in bed format using the ‘getfasta’ command of bedtools with the ‘s’ option enabled. Individual exon sequences are then merged, creating a single contiguous 3′-UTR sequence. FilTar then converts miRNA and 3′-UTR sequence and identifier information to a format which can be parsed by TargetScan algorithms.

TargetScan is executed using both Ensembl 3′-UTR annotations, and updated annotations produced using FilTar for the purposes of the differential expression analyses reported in this study.

The FilTar tool is also fully compatible with the miRanda (v3.3a) (Enright *et al.*, 2003; John *et al.*, 2004) miRNA target prediction algorithm allowing users to identify non-canonical miRNA targets, that is predicted targets without a perfectly complementary seed match to the miRNA.

### 2.5 Transcript quantification

Human and mouse cDNA files were downloaded from Ensembl. Kallisto (v0.44.0) (Bray *et al.*, 2016) was used to index the cDNA data using the ‘kallisto index’ command with default parameters. Reads were pseudoaligned and relative transcript abundance quantified using the ‘kallisto quant’ executable, using the ‘bias’ option to correct for sequence-based biases. When kallisto was used with data derived from single-end RNA-sequencing experiments, 180 and 20 nt were used as required estimates of the mean average fragment length and SD, respectively.

### 2.6 Availability of data and materials

See Supplementary Methods for information regarding the selection and analysis of data used in this article. All data analysed in this study are publicly available and a table of relevant project accessions is given (Supplementary Table S1), along with relevant QC statistics (Supplementary Table S2). The FilTar tool is publicly and freely accessible for download (https://github.com/TBradley27/FilTar) with full supporting documentation (https://tbradley27.github.io/FilTar/).

## 3 Results

In order to benchmark the performance of the FilTar tool in a specific cellular context versus general miRNA target prediction we used RNA-Seq data from miRNA mimic transfection experiments in mouse and human cell lines. Fold change values represent changes in relative mRNA abundance in samples transfected with a miRNA mimic compared to samples transfected with a negative control.

### 3.1 Expression filtering

**Fig. 1.**
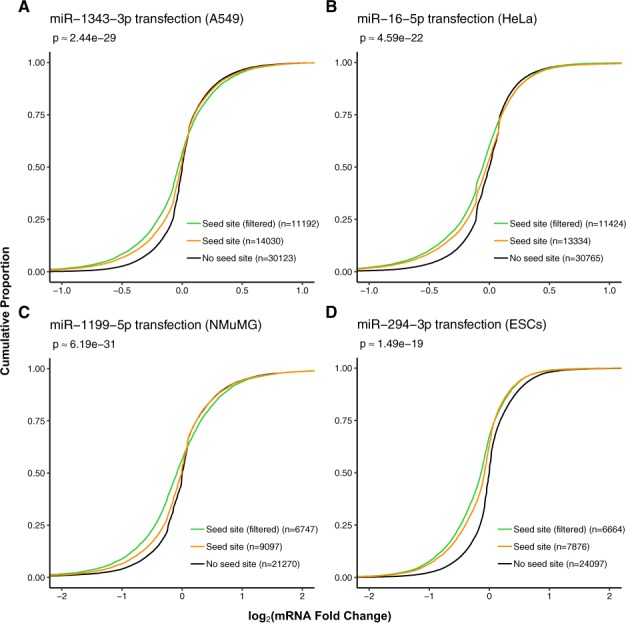
Implementing an expression threshold on predicted miRNA targets improves miRNA target prediction accuracy. Results are derived from miRNA mimic and control transfection experiments. Curves show the cumulative log_2_ fold change distributions of: (i) protein-coding non-target transcripts (black), (ii) protein-coding seed target transcripts (orange) and (iii) expression filtered (TPM ≥ 0.1) protein-coding seed target transcripts (green). Numbers in round brackets represent the number of mRNA transcripts contained in each distribution. Approximate *P*-values were computed using one-sided, two-sample, Kolmogorov–Smirnov tests between unfiltered and filtered target fold change distributions. Data presented for miRNA mimic transfection into (**A**) A549 and (**B**) HeLa cell lines, (**C**) normal murine mammary gland (NMuMG) cells and (**D**) mouse embryonic stem cells (ESCs)

Predicted miRNA targets which were filtered according to their expression level, at a TPM (transcripts per million) (Li *et al.*, 2009) threshold of 0.1, as a whole, exhibited stronger repression after miRNA transfection than the full miRNA target set without expression filtering (Fig. 1 and Supplementary Fig. S1). Predicted miRNA targets removed by FilTar generally exhibited low absolute fold change values suggesting that these are false-positive predictions in these specific cellular contexts (Supplementary Fig. S2). Implementing expression filters for a range of different TPM values reveals that increasing this threshold results in a stronger filtering effect on retained mRNAs (Supplementary Fig. S3a). However, increasing the expression threshold beyond a particular point (between 1 and 10 TPM for experiments analysed) leads to the removal of a considerable number of mRNA transcripts which are repressed by the transfection of a miRNA mimic (Supplementary Fig. S3b).

The number and percentage of annotated protein-coding transcripts which are used in FilTar’s 3′-UTR reannotation workflow, for each sample after expression filtering, are given in Supplementary Table S3. Only those transcripts possessing a pre-existing 3′-UTR annotation spanning only a single exon are selected (see Materials and methods).

### 3.2 3′-UTR extension

**Fig. 2.**
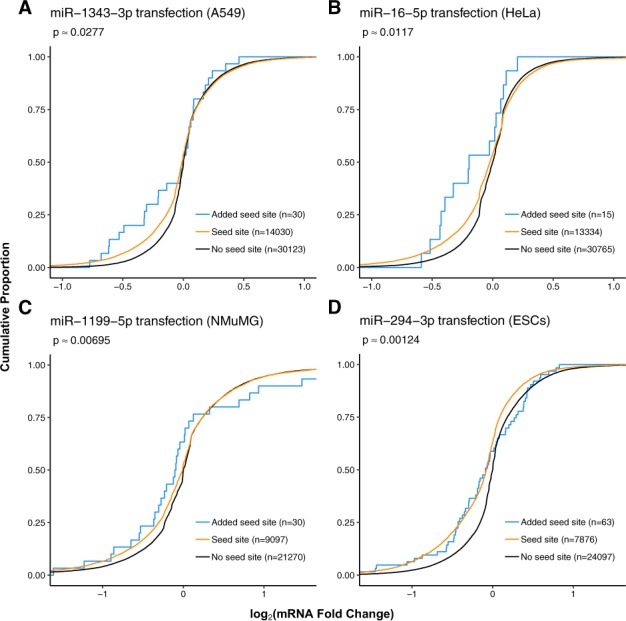
3′-UTR elongation by FilTar leads to the identification of additional valid miRNA targets. Curves show the cumulative log_2_ fold change distributions of: (i) protein-coding non-target transcripts (black), (ii) protein-coding seed target transcripts (orange) and (iii) predicted target transcripts deriving from FilTar 3′-UTR annotations but not Ensembl 3′-UTR annotations (blue). Approximate *P*-values were computed using one-sided, two-sample, Kolmogorov–Smirnov tests between pre-existing target and newly identified target fold change distributions. Otherwise as in Figure 1

Newly gained miRNA target predictions deriving from FilTar’s refined 3′-UTR annotations of protein-coding transcripts (*i.e.* miRNA targets deriving from the elongation of existing 3′-UTR annotations), generally exhibited similar levels of repression to miRNA target predictions deriving from Ensembl 3′-UTR annotations (Fig. 2 and Supplementary Fig. S4). Anomalies were results deriving from the transfection of miR-107 and miR-10a-5p miRNA mimics into HeLa cells in which newly identified miRNA target predictions did not exhibit a log fold change distribution commensurate with that exhibited by already existing miRNA target predictions (Supplementary Fig. S4).

### 3.3 3′-UTR truncation

**Fig. 3.**
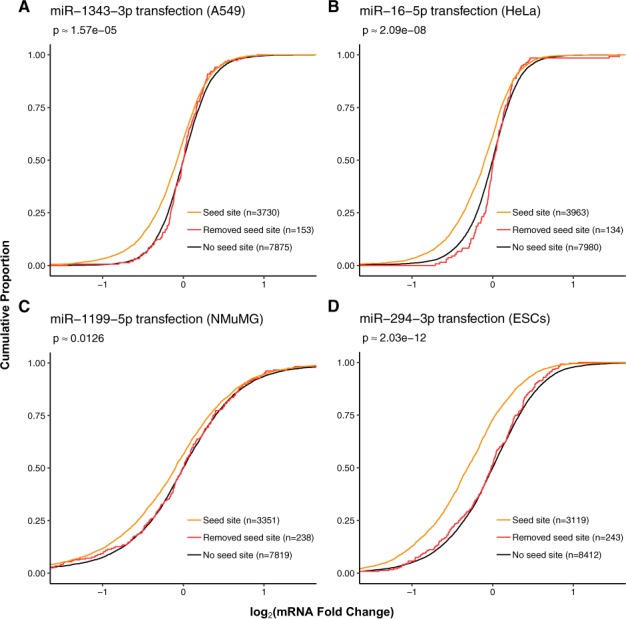
3′-UTR truncation by FilTar leads to the removal of false-positive miRNA target predictions. Curves are plotted of the cumulative log_2_ fold change distributions of expression filtered: (i) protein-coding non-target transcripts (black), (ii) protein-coding seed target transcripts (orange) and (iii) predicted target transcripts deriving from Ensembl 3′-UTR annotations but not FilTar 3′-UTR annotations (red). Approximate *P*-values were computed using one-sided, two-sample, Kolmogorov–Smirnov tests between non-target and discarded miRNA target fold change distributions. Otherwise as in Figure 1

Conversely, miRNA target transcripts that were removed as a result of FilTar truncating 3′-UTR annotations relative to standard Ensembl annotations, exhibited repression similar to that of annotated non-target transcripts (Fig. 3 and Supplementary Fig. S5). In a minority of datasets analysed, removed target transcripts exhibited significantly less repression than target transcripts, but nonetheless exhibited greater repression than annotated non-target transcripts. In these datasets, the removed target log fold change distribution tended to align with the non-target distribution at the negative extremity, but not at small negative fold change value ranges—indicating that for a minority of datasets, labelled ‘removed targets’ may be mildly repressed by targeting miRNAs. Additional analysis demonstrated that for these datasets, such targets exhibited significantly weaker repression in response to miRNA transfection than 6-mer targets, which are the weakest canonical miRNA target site type (Bartel, 2018) (Supplementary Fig. S6).

### 3.4 Cumulative effect of filtering and reannotation

**Fig. 4.**
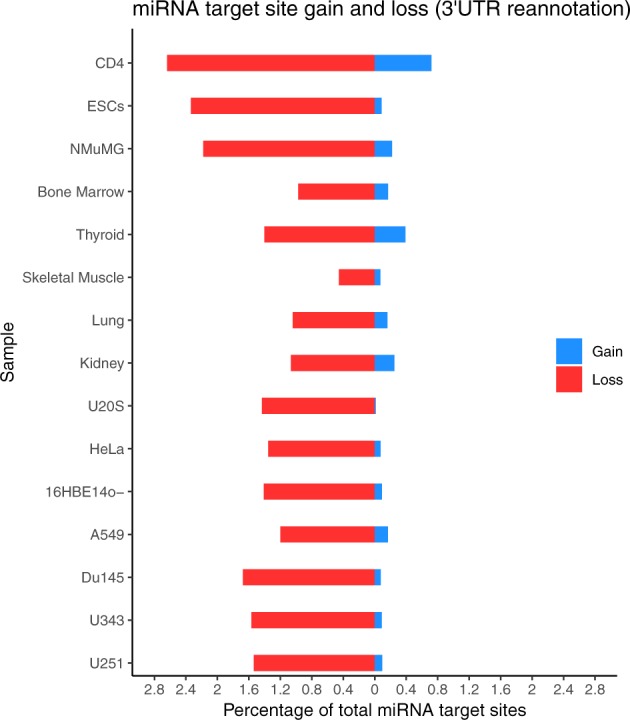
Total miRNA target site gain and loss when applying FilTar to multiple sample types. FilTar is applied to the protein-coding transcriptome for all annotated human miRNAs for multiple tissues, organs and cell lines. Gained (blue) and lost (red) miRNA target sites are expressed as a percentage of the total number of target sites identified when deriving miRNA from Ensembl 3′-UTR annotations

When the FilTar reannotation and miRNA target prediction workflow was applied transcriptome-wide, to multiple organs and cell lines, using all annotated miRBase human miRNAs, there was a mean average gain and loss of miRNA target sites corresponding to 0.18% and 1.5% of the total original miRNA target sites predicted deriving from Ensembl 3′-UTR annotations (Fig. 4). This corresponds to a gain and loss of total miRNA seed sides in the tens and hundreds of thousands, respectively (Supplementary Table S4). Although a much larger proportion of miRNA seed sites (mean average of 26.3%) are lost through expression filtering (Supplementary Fig. S7), representing a loss of millions of miRNA seed sites (Supplementary Table S4). This is commensurate with the mean average of 34.0% of 3′-UTR bases lost when removing lowly expressed transcripts (<0.1 TPM) from target predictions (Supplementary Table S5), which is greater than the mean average of 2.0% of bases lost through 3′-UTR reannotation (Supplementary Table S6). When considering the combined effect of expression filtering and 3′-UTR reannotation, a mean average 36.1% of 3′-UTR bases are lost, affecting a mean average of 53.4% of protein-coding 3′-UTRs (Supplementary Table S7).

## 4 Discussion

Results show that FilTar is successfully able to utilize RNA-Seq data to reannotate protein-coding 3′-UTR sequences and filter based on expression data leading to a gain in specificity and sensitivity of target prediction evidenced through tests using experimental data.

Expression filtering target transcripts at even a modest expression threshold of 0.1 TPM leads to a loss of millions of seed sites in most datasets analysed (Supplementary Table S4), representing a radical reduction in the number of false-positive predictions associated with miRNA target prediction. This is indicative of the importance of considering the biological plausibility of candidate miRNA interactions. The positive relationship between the expression threshold chosen and the extent of repression of retained mRNA transcripts is evidence for the robustness of this effect (Supplementary Fig. S3a). The increase in specificity conferred by expression filtering does, however, seem to be accompanied by a corresponding loss of sensitivity of miRNA target prediction when large expression threshold values are chosen (Supplementary Fig. S3b), indicating that sufficient caution ought to be exercised by the user when choosing expression threshold values. However, even for larger expression thresholds, the reduction in sensitivity is less than the increase in specificity conferred by expression filtering (Supplementary Fig. S3a).

The number of newly predicted miRNA target sites deriving from FilTar elongated 3′-UTR sequences is generally relatively low. For cell line datasets analysed, the maximum of number of newly predicted miRNA targets made for any single miRNA was 63, with the majority of datasets analysed yielding less than 15 newly predicted targets (Fig. 2 and Supplementary Fig. S4). The number of newly identified target transcripts is commensurate with the universally low proportion of 3′-UTRs extended, and the small proportion of bases added to the total of the 3′-UTR annotation (Supplementary Table S6), even though this still represents a substantial increase in the number of miRNA seed target sites identified. This is in contrast to 3′-UTR truncation in which the proportion of 3′-UTRs truncated and bases removed from the 3′-UTR annotation total are much greater. Analysis shows that there is a strong positive correlation between the number of 3′-UTR bases reannotated, and the number of predicted miRNA target sites gained or lost through reannotation (Supplementary Fig. S8a and b). The bias in 3′-UTR truncation as opposed to elongation can possibly be explained by either a pre-existing bias in standard Ensembl 3′-UTR annotations to generate long 3′-UTR models, or rather a bias in the FilTar reannotation workflow for 3′-UTR truncation rather than elongation. A potential bias in the standard Ensembl annotation workflow could potentially be explained by the method of transcript annotation, in which, although transcript models are built on a tissue-specific basis, transcript models incorporated into the final Ensembl gene set typically only derive from the merging of RNA-sequencing reads from multiple different tissue samples (Aken *et al.*, 2016), therefore, creating a bias towards the annotation of longer 3′-UTRs. This effect may be exacerbated or supplemented by the existence of 3′-UTR isoforms within a given sample and transcript—creating relatively low abundance isoforms towards the distal end of the 3′-UTR, making annotation difficult and likely generating a large amount of uncertainty, biases and variability in different methods used to model 3′-UTRs.

Another possibility is that the shortening and extension of existing 3′-UTR annotations are qualitatively different problems requiring different respective sequencing depths. Within a given sample, a read sampling analysis demonstrates that there is a positive relationship, up to a point of saturation between sequencing depth and the number of bases used to elongate existing 3′-UTRs (Supplementary Fig. S9a). In addition, the saturation point for the addition of bases to 3′-UTRs is still substantially less than the proportion of bases removed at 3′-UTRs even at relatively low sequencing depths indicating that the discrepancy between proportion of 3′-UTR bases added or subtracted from the 3′-UTRs cannot be explained by insufficient sequencing depth. A similar positive relationship is observed between sequencing depth and the number of based truncated from existing 3′-UTRs (Supplementary Fig. S9b), although far fewer reads seem to be required for saturation to occur, indicating a weaker reliance on sequencing depth for 3′-UTR truncation compared to 3′-UTR elongation.

Although as mentioned previously, the sequencing depth does seem to influence the extent of 3′-UTR reannotation, for a set of different biological samples, sequencing depth alone seems to have limited predictive value for this variable (Supplementary Fig. S10a and b). The likely explanation being that as well as sequencing depth, the extent of 3′-UTR reannotation is also determined by other key variables such as the cell type being analysed, read length used for sequencing, library preparation protocol, the use of single-end or paired-end sequencing, as well as additional researcher or lab-specific batch effects (Leek *et al.*, 2010). For example, as some cell types are biased towards shorter 3′-UTRs (Mayr and Bartel, 2009), while others towards longer 3′-UTRs (Miura *et al.*, 2013), generating radically different reannotation statistics irrespective of sequencing depth used.

As mentioned previously, there was generally a much larger number of miRNA target sites predicted to be removed than added during 3′-UTR reannotation. This is despite FilTar permitting 3′-UTR truncations only occurring on moderately-to-highly expressed transcripts, after discovery that the reannotation of the 3′-UTRs of lowly expressed transcripts generated a relatively large number of what seemed to be false-positive predictions (Supplementary Fig. S11). The likely cause being that low transcript expression leads to sporadic and inconsistent coverage across the 3′-UTR, in which there is insufficient information to correctly call 3′-UTR truncation. The default behaviour of the FilTar tool therefore is to only truncate the 3′-UTRs of transcripts which are not poorly expressed (*i.e.* TPM ≥ 5).

When examining 3′-UTR truncations further, for a minority of datasets analysed, some removed predicted miRNA targets seem to be marginally effective, with some transcripts exhibiting low levels of repression upon transfection of the miRNA mimic. Further analysis indicates that these marginally repressed transcripts exhibit even weaker repression than 6-mer targeted transcripts (Supplementary Fig. S6), one of the least effective canonical miRNA target types (Bartel, 2018), indicating that the efficacy of these site types is marginal. A possible explanation for the existence of these site types is that, for some transcript annotations for which the 3′-UTR was truncated, there may exist a small proportion of isoforms with longer 3′-UTRs, which are too low in abundance to be detected by APAtrap, but nonetheless still confer a marginal level of repression to the transcript, and hence is detectable when analysing experimental data.

Investigations into the effect of utilising expression data when making transcriptome-wide miRNA target predictions can be extended by closer examination of not only the refinement of 3′-UTR annotations across different biological contexts, and its effects on miRNA target prediction, but more precisely the definition of specific 3′-UTR profiles, incorporating information about 3′-UTR isoforms within a given cellular context (Agarwal *et al.*, 2015). This enables the weighting of miRNA target prediction scores on the basis of sequencing data applied by the user themselves, enabling even further and extended tailoring of miRNA target prediction to the specific biological context being researched. Previous analyses indicate that the most effective target predictions occur when those predictions are weighted on the basis of 3′-UTR isoform ratios (Nam *et al.*, 2014). In addition, the scope of FilTar’s functionality can be increased by enabling the annotation of novel 3′-UTR sequences for transcripts without a current annotated 3′-UTR, and also for those 3′-UTRs which themselves span multiple exons. In addition, both the configurability and precision of FilTar can be improved in the future by, respectively, enabling use of additional tools for 3′-UTR reannotation (Gruber *et al.*, 2018a, b) and exploring the greater transcriptomic resolutions enabled by nascent single cell sequencing technologies.

## 5 Conclusion

FilTar utilizes RNA-Seq data to increase the accuracy of miRNA target predictions in animals by filtering for expressed mRNA transcripts and reannotating 3′-UTRs for greater specificity to a given cellular context of interest to the researcher. FilTar’s compatibility with user-generated RNA-Seq data confers functionality across a wide range of potential biological contexts.

## Supplementary Material

btaa007_Supplementary_Data
