# Supplementary material for: FilTar: using RNA-Seq data to improve microRNA target prediction accuracy in animals
Source: Bioinformatics. 2020 Jan 13;36(8):2410–6. doi: 10.1093/bioinformatics/btaa007 (PMC7178423; doi:10.1093/bioinformatics/btaa007)
Supplement: btaa007_Supplementary_Data [file btaa007_supplementary_data.pdf]

Supplementary Information for:

FilTar: Using RNA-Seq data to improve microRNA target prediction accuracy in animals

Thomas Bradley[1,2] and Simon Moxon[1]

Affiliations:

1. School of Biological Sciences, University of East Anglia, Norwich NR4 7TJ, UK

2. Earlham Institute, Norwich Research Park, Norwich NR4 7UZ, UK

Corresponding Author: Simon Moxon (s.moxon@uea.ac.uk)

Table of Contents

Supplementary Methods

Data Selection......4

Quality control and statistics......4

Differential expression analysis......5

Data visualisation......5

References......6

Supplementary Figures

S1: As in Figure 1, though with a greater number of datasets analysed......10

S2: Analysis of potential mRNA targets removed by expression filtering......13

S3a: The effect of expression filtering on potential mRNA targets at multiple threshold......14

S3b: As in Supplementary Figure S2, though with multiple expression thresholds......15

S4: As in Figure 2, though with a greater number of datasets analysed......16

S5: As in Figure 3, though with a greater number of datasets analysed......17

S6: Analysis of the targeting efficacy of potential FilTar false negatives......20

S7: Total miRNA target site loss as a result of expression filtering......21

S8a: The relationship between 3'UTR elongation and predicted miRNA target sites gained......22

S8b: The relationship between 3'UTR truncation and predicted miRNA target sites lost......23

S9a: The within-sample relationship between 3'UTR elongation and sequencing depth......24

S9b: The within-sample relationship between 3'UTR truncation and sequencing depth......25

S10a: The between-sample relationship between 3'UTR elongation and sequencing depth......26

S10b: The between-sample relationship between 3'UTR truncation and sequencing depth......27

S11: An analysis of 3'UTR predicted miRNA target without additional expression filtering......28

Supplementary Tables

S1: Number and percentage of 3'UTRs with pre-existing 3'UTR annotations......30

S2: Total predicted miRNA target sites gained and using FilTar......31

S3: Loss of 3'UTR bases as a result of expression filtering......35

S4: Gain and loss of 3'UTR bases as a result of 3'UTR reannotation......36

S5: Total loss of 3'UTR bases aggregating data from Supplementary Tables S1 and S2......37

S6: Metadata of publicly available datasets used in this study......38

S7: QC statistics for all datasets used in the analysis......39

Supplementary Methods

Data selection

For analysis of miRNA transfection experiments, FASTQ sequencing data generated from RNA-Seq experiments in human or mouse cell lines with at least two biological replicates were selected for further processing. It is expected that samples transfected with a specific miRNA would lead to a reduction in expression of its target relative to the control sample. After differential expression analysis, if by inspection of cumulative plots, the predicted miRNA targets could not be observed to be downregulated relative to non-target transcripts, then the transfection experiment was considered to have failed, and relevant datasets were not used for downstream analysis.

A summary of datasets used with relevant database accessions is provided (Supplementary Table S1) (1-8).

For subsampling experiments shown in Supplementary Figures S9a and S9b, total reads were sampled using the seqtk tool (https://github.com/lh3/seqtk).

Quality control and statistics

FASTQ data quality scores, GC-content, read lengths and similar statistics were generated using FASTQC (v0.11.5) (https://www.bioinformatics.babraham.ac.uk/projects/fastqc/). Output from FASTQC was collated with data from the log files of other processes in order to produce a summary statistics report for each used BioProject using MultiQC (v1.6) (9) (output summarised in Supplementary Table S2).

Differential expression analysis

Differential expression analysis for miRNA transfection experiments was completed within the R (v.3.5.0) statistical computing environment (10). Transcript-level read count data derived from RNA sequencing of miRNA mimic or negative control transfected cell lines were imported using the tximport package (v1.10.1) (11). Differential expression analysis on length and library size normalised read counts was performed using DESeq2 (v1.22.2) (12) comparing expression between negative control and miRNA mimic transfection conditions. Log_2 fold change values were subsequently shrunken using the default DESeq2 'normal' shrinkage estimator (12) to account for the large uncertainty in predicted fold change values at low transcript expression values. For plotting, records corresponding to non-coding RNA transcripts were discarded. Transcript records were discarded when there was zero expression for all control and transfection replicates and fold change values could not be calculated. Target prediction data was used to label the remaining records as either predicted targets or non-targets of the transfected miRNA.

For some differential expression analyses, null hypothesis significance testing was performed using two-sample, one-sided Kolmogorov-Smirnov tests to test whether different fold change distributions were sampled from the same underlying distribution.

Data Visualisation

All visualisations are produced using R's ggplot2 package (v3.1.0) (13).

For Figure 1, the filtered miRNA predicted targets curves represents protein-coding transcripts with a miRNA seed target site to the transfected miRNA mimic, which have been filtered at an expression threshold of 0.1 *transcripts per million* (TPM) (14).

For Figure 2, the ‘added seed sites’ are identified as those transcripts which had not previously been labelled as predicted miRNA targets using target prediction results derived from existing Ensembl 3’UTR annotations, but had been identified as predicted miRNA targets using target prediction results derived from 3’UTR sequences reannotated using the FilTar workflow due to 3’UTR extension.

For Figure 3, the ‘removed seed sites’ are identified as those transcripts which had previously been labelled as predicted miRNA targets using target prediction results derived from existing Ensembl 3’UTR annotations, but had not been identified as predicted miRNA targets using target prediction results derived from 3’UTR sequences reannotated using the FilTar workflow due to 3’UTR truncation. Filtering for all groups occurred at an expression threshold of greater than or equal to 5 TPM. This was to reduce the number of false positive 3’UTR truncations (see discussion).

Additional plots for remaining datasets analysed are contained within Supplementary Figures S1, S4 and S5 with the exception of cases were there was an insufficient number of added or removed target transcripts predicted (n < 15).

References

1. Tamim S, Vo DT, Uren PJ, Qiao M, Bindewald E, Kasprzak WK, et al. Genomic analyses reveal broad impact of miR-137 on genes associated with malignant transformation and neuronal differentiation in glioblastoma cells. PloS one. 2014;9(1):e85591.

2. Liu C, Liu R, Zhang D, Deng Q, Liu B, Chao H-P, et al. MicroRNA-141 suppresses prostate cancer stem cells and metastasis by targeting a cohort of pro-metastasis genes. Nature communications. 2017;8:14270.

3. Stolzenburg LR, Wachtel S, Dang H, Harris A. miR-1343 attenuates pathways of fibrosis by targeting the TGF-β receptors. Biochemical Journal. 2016;473(3):245-56.

4. Liu W, Wang X. Prediction of functional microRNA targets by integrative modeling of microRNA binding and target expression data. Genome biology. 2019;20(1):18.

5. Guo JU, Agarwal V, Guo H, Bartel DP. Expanded identification and characterization of mammalian circular RNAs. Genome biology. 2014;15(7):409.

6. Diepenbruck M, Tiede S, Saxena M, Ivanek R, Kalathur RKR, Lüönd F, et al. miR-1199-5p and Zeb1 function in a double-negative feedback loop potentially coordinating EMT and tumour metastasis. Nature communications. 2017;8(1):1168.

7. Pua HH, Steiner DF, Patel S, Gonzalez JR, Ortiz-Carpena JF, Kageyama R, et al. MicroRNAs 24 and 27 suppress allergic inflammation and target a network of regulators of T helper 2 cell-associated cytokine production. Immunity. 2016;44(4):821-32.

8. Cao Y, Guo WT, Tian S, He X, Wang XW, Liu X, et al. miR-290/371-Mbd2-Myc circuit regulates glycolytic metabolism to promote pluripotency. The EMBO journal. 2015;34(5):609-23.

9. Ewels P, Magnusson M, Lundin S, Käller M. MultiQC: summarize analysis results for multiple tools and samples in a single report. Bioinformatics. 2016;32(19):3047-8.

10. Team RC. R: A language and environment for statistical computing. 2013.

11. Soneson C, Love MI, Robinson MD. Differential analyses for RNA-seq: transcript-level estimates improve gene-level inferences. F1000Research. 2015;4.

12. Love MI, Huber W, Anders S. Moderated estimation of fold change and dispersion for RNA-seq data with DESeq2. Genome biology. 2014;15(12):550.

13. Wickham H. ggplot2: elegant graphics for data analysis: Springer; 2016.

14. Li B, Ruotti V, Stewart RM, Thomson JA, Dewey CN. RNA-Seq gene expression estimation with read mapping uncertainty. Bioinformatics. 2009;26(4):493-500.

Supplementary Figures

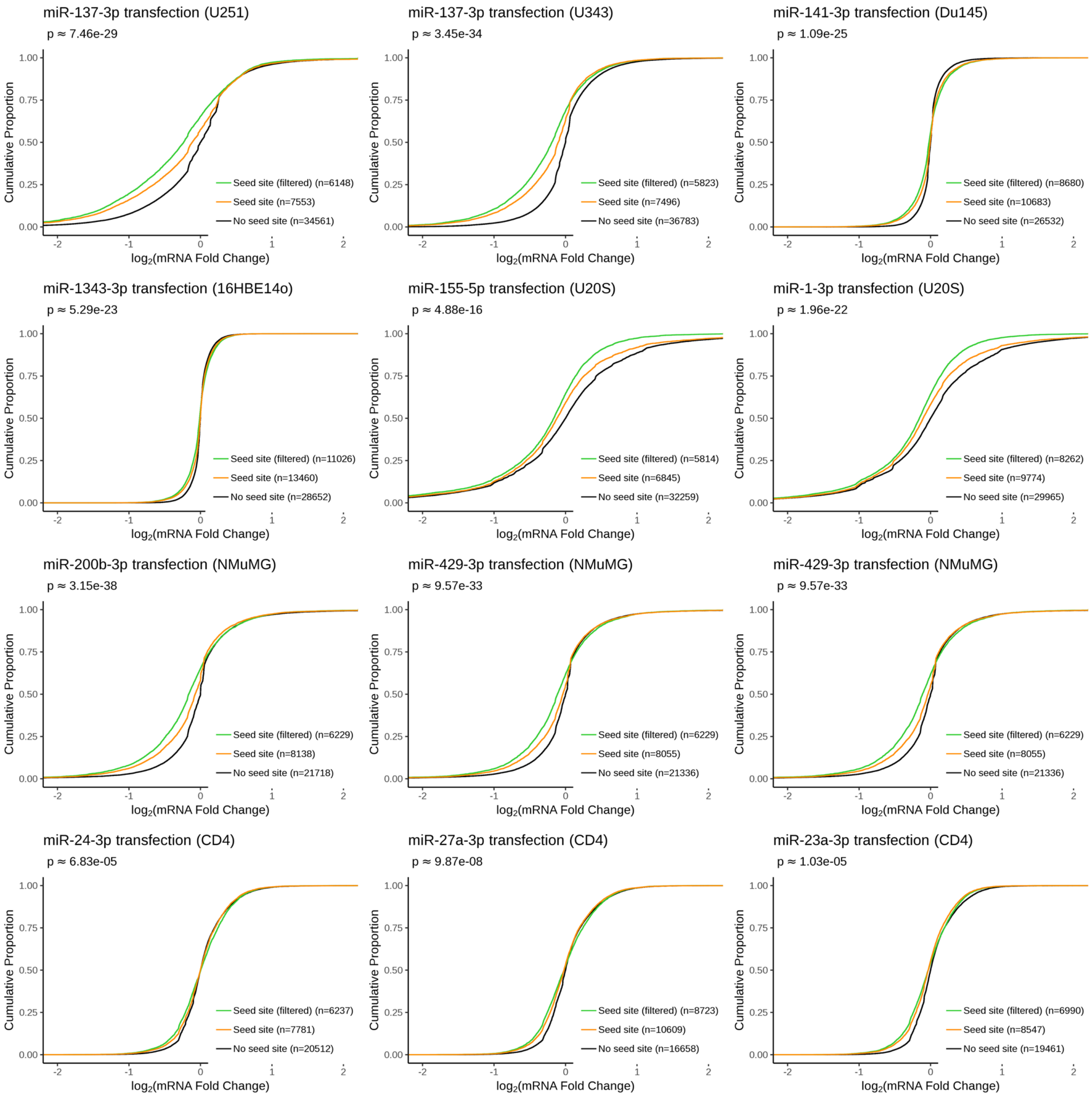
miR-137-3p transfection (U251)
p ≈ 7.46e-29
Seed site (filtered) (n=6148)
Seed site (n=7553)
No seed site (n=34561)
miR-137-3p transfection (U343)
p ≈ 3.45e-34
Seed site (filtered) (n=5823)
Seed site (n=7496)
No seed site (n=36783)
miR-141-3p transfection (Du145)
p ≈ 1.09e-25
Seed site (filtered) (n=8680)
Seed site (n=10683)
No seed site (n=26532)
miR-1343-3p transfection (16HBE14o)
p ≈ 5.29e-23
Seed site (filtered) (n=11026)
Seed site (n=13460)
No seed site (n=28652)
miR-155-5p transfection (U20S)
p ≈ 4.88e-16
Seed site (filtered) (n=5814)
Seed site (n=6845)
No seed site (n=32259)
miR-1-3p transfection (U20S)
p ≈ 1.96e-22
Seed site (filtered) (n=8262)
Seed site (n=9774)
No seed site (n=29965)
miR-200b-3p transfection (NMuMG)
p ≈ 3.15e-38
Seed site (filtered) (n=6229)
Seed site (n=8138)
No seed site (n=21718)
miR-429-3p transfection (NMuMG)
p ≈ 9.57e-33
Seed site (filtered) (n=6229)
Seed site (n=8055)
No seed site (n=21336)
miR-429-3p transfection (NMuMG)
p ≈ 9.57e-33
Seed site (filtered) (n=6229)
Seed site (n=8055)
No seed site (n=21336)
miR-24-3p transfection (CD4)
p ≈ 6.83e-05
Seed site (filtered) (n=6237)
Seed site (n=7781)
No seed site (n=20512)
miR-27a-3p transfection (CD4)
p ≈ 9.87e-08
Seed site (filtered) (n=8723)
Seed site (n=10609)
No seed site (n=16658)
miR-23a-3p transfection (CD4)
p ≈ 1.03e-05
Seed site (filtered) (n=6990)
Seed site (n=8547)
No seed site (n=19461)
Cumulative Proportion
log2(mRNA Fold Change)
0.00
0.25
0.50
0.75
1.00
-2
-1
0
1
2

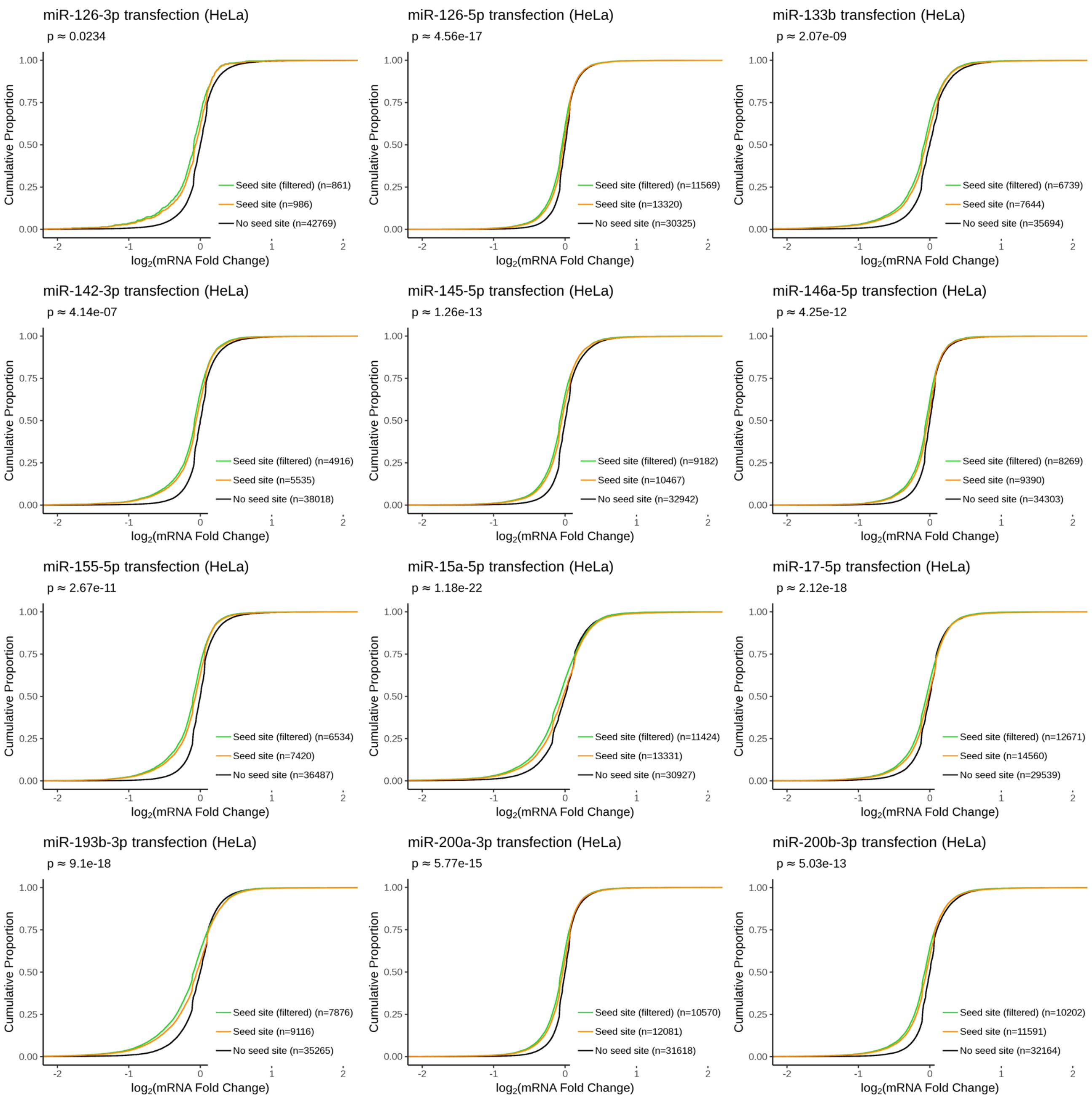
miR-126-3p transfection (HeLa)
p ≈ 0.0234
Seed site (filtered) (n=861)
Seed site (n=986)
No seed site (n=42769)
miR-126-5p transfection (HeLa)
p ≈ 4.56e-17
Seed site (filtered) (n=11569)
Seed site (n=13320)
No seed site (n=30325)
miR-133b transfection (HeLa)
p ≈ 2.07e-09
Seed site (filtered) (n=6739)
Seed site (n=7644)
No seed site (n=35694)
miR-142-3p transfection (HeLa)
p ≈ 4.14e-07
Seed site (filtered) (n=4916)
Seed site (n=5535)
No seed site (n=38018)
miR-145-5p transfection (HeLa)
p ≈ 1.26e-13
Seed site (filtered) (n=9182)
Seed site (n=10467)
No seed site (n=32942)
miR-146a-5p transfection (HeLa)
p ≈ 4.25e-12
Seed site (filtered) (n=8269)
Seed site (n=9390)
No seed site (n=34303)
miR-155-5p transfection (HeLa)
p ≈ 2.67e-11
Seed site (filtered) (n=6534)
Seed site (n=7420)
No seed site (n=36487)
miR-15a-5p transfection (HeLa)
p ≈ 1.18e-22
Seed site (filtered) (n=11424)
Seed site (n=13331)
No seed site (n=30927)
miR-17-5p transfection (HeLa)
p ≈ 2.12e-18
Seed site (filtered) (n=12671)
Seed site (n=14560)
No seed site (n=29539)
miR-193b-3p transfection (HeLa)
p ≈ 9.1e-18
Seed site (filtered) (n=7876)
Seed site (n=9116)
No seed site (n=35265)
miR-200a-3p transfection (HeLa)
p ≈ 5.77e-15
Seed site (filtered) (n=10570)
Seed site (n=12081)
No seed site (n=31618)
miR-200b-3p transfection (HeLa)
p ≈ 5.03e-13
Seed site (filtered) (n=10202)
Seed site (n=11591)
No seed site (n=32164)
Cumulative Proportion
log2(mRNA Fold Change)

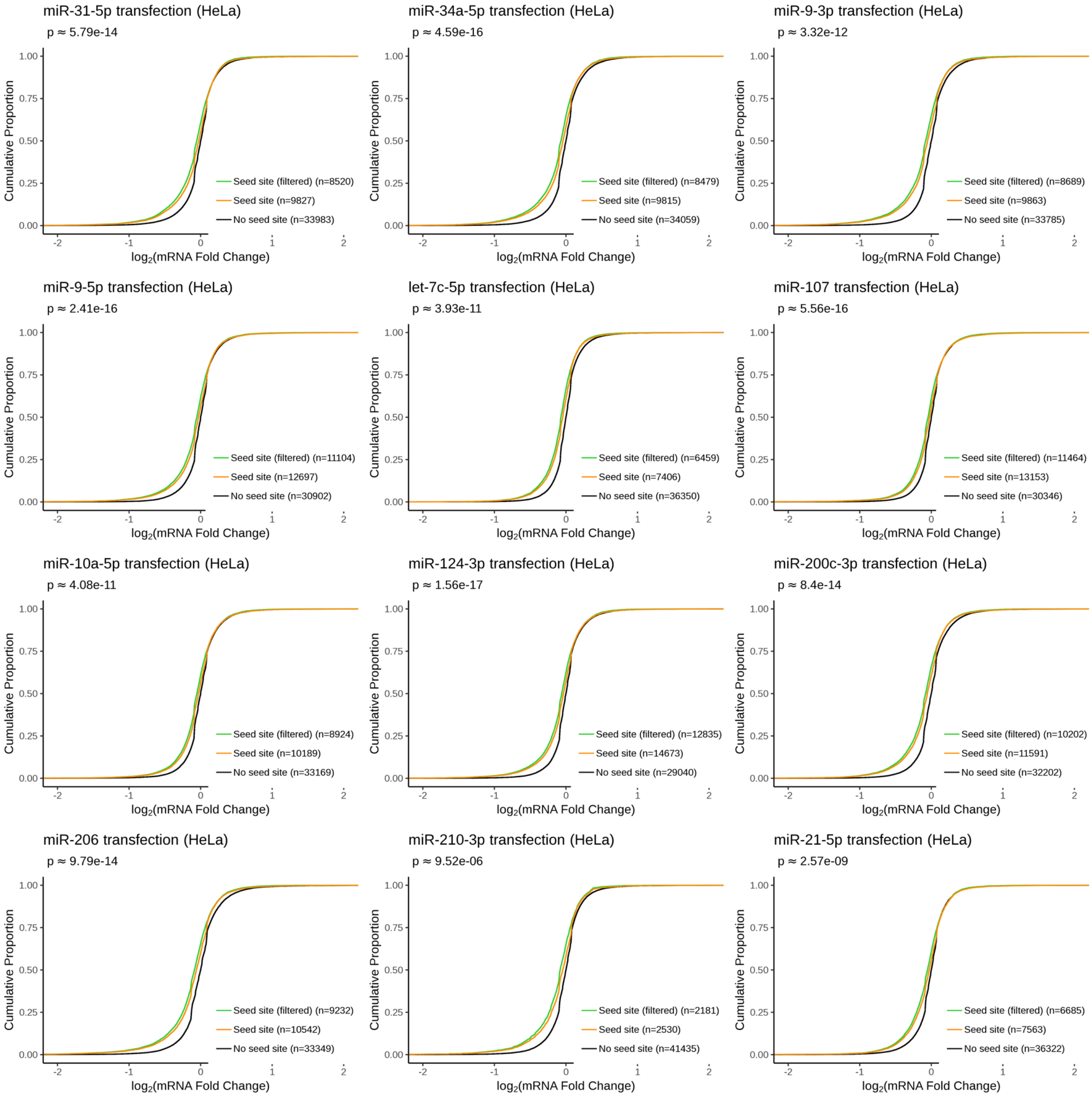

Supplementary Fig. S1 - As in Figure 1, though with additional datasets analysed

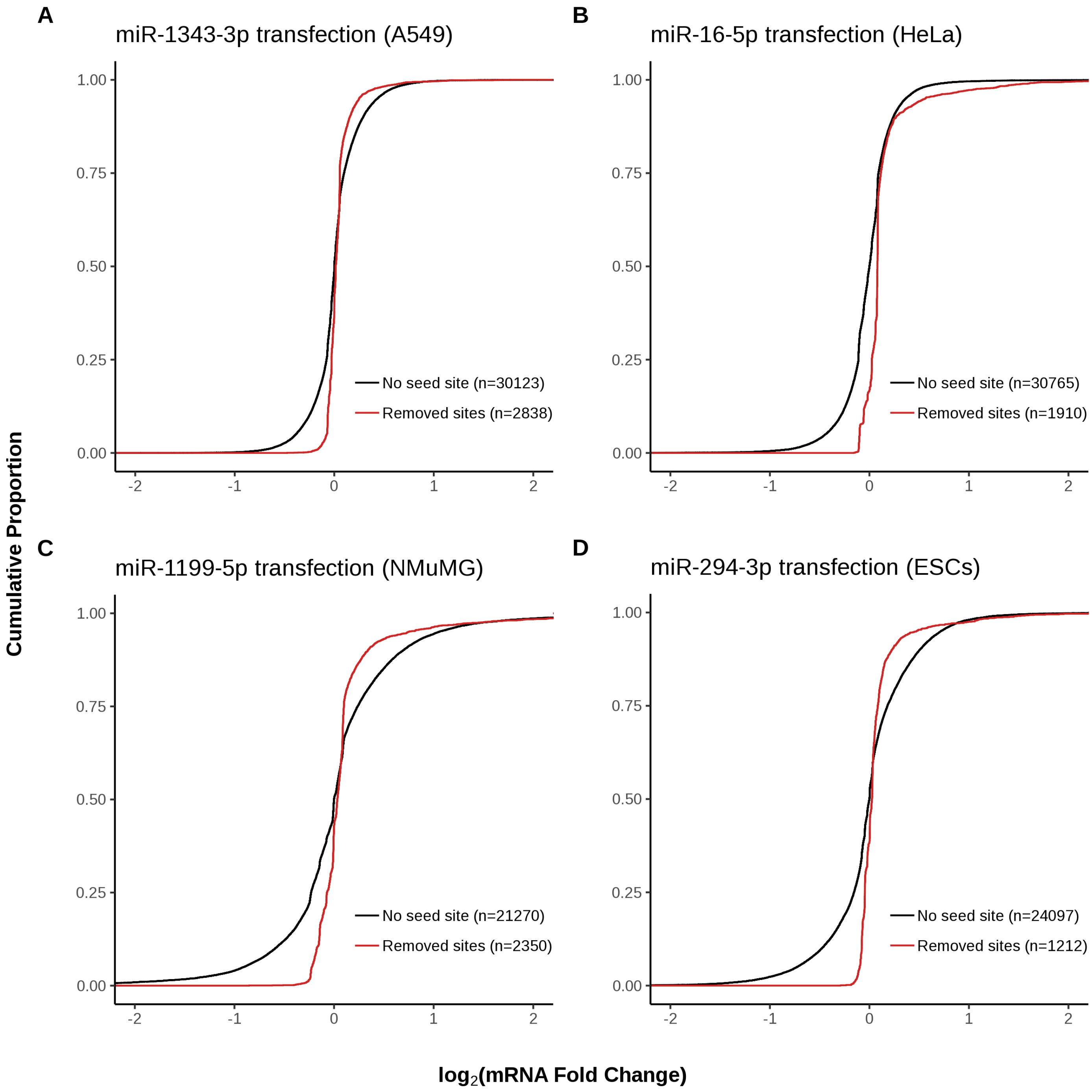

Supplementary Fig. S2: For the analysis presented in Figure 1, the cumulative $\log_2$ fold change distributions of lowly expressed transcripts (<0.1 TPM) with canonical seeds sites (dark red), in their 3'UTRs compared against the distribution of transcripts without a canonical seed site in their 3'UTRs (black).

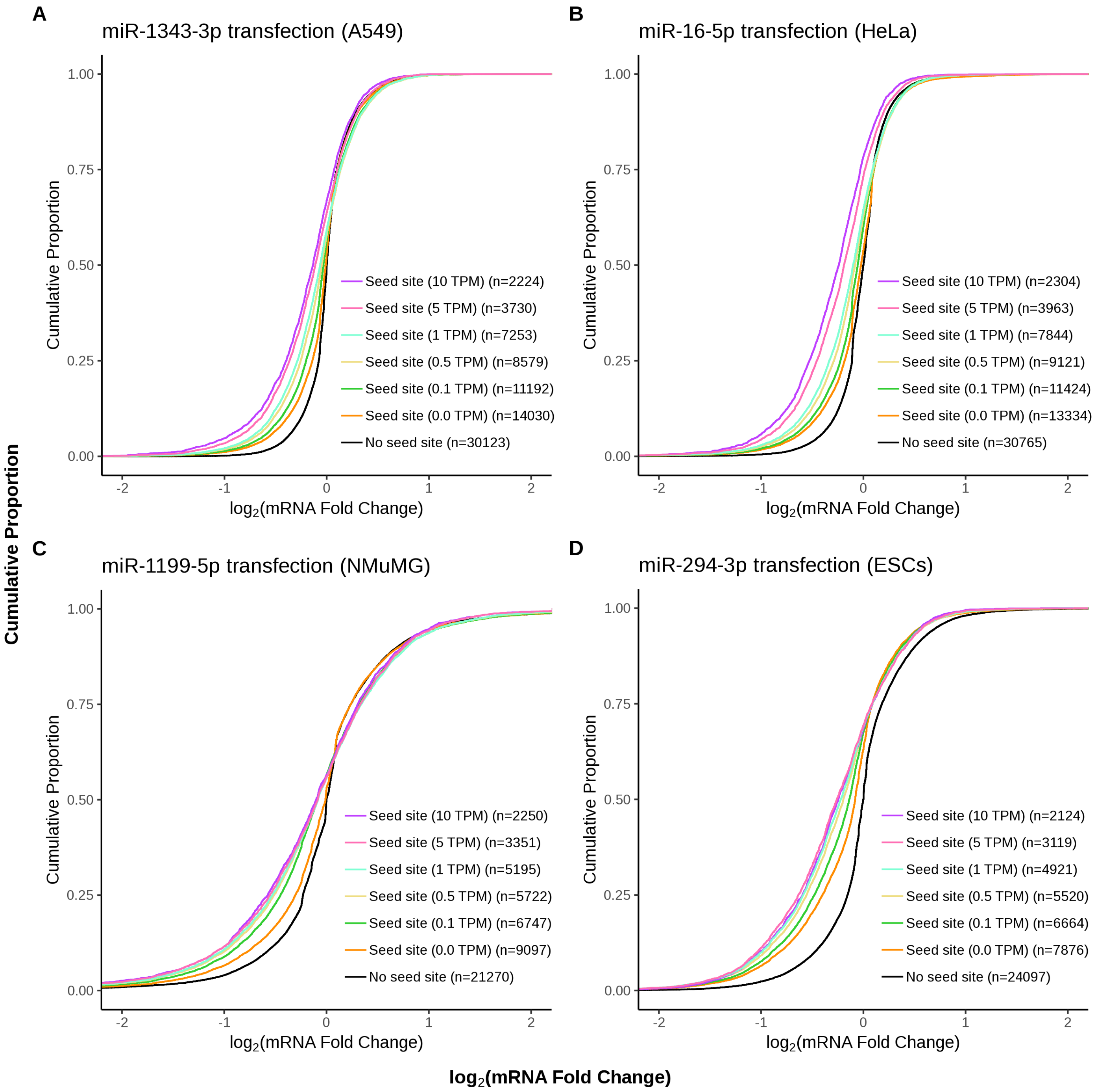

Supplementary Fig. S3a: The effect of expression filtering on retained protein-coding transcripts using multiple expression thresholds. Expression thresholds are implemented at TPM values of 10 (purple), 5 (pink), 1 (light blue), 0.5 (gold), 0.1 (green) and 0.0 (orange). Otherwise as in Figure 1 and Supplementary Figure S1.

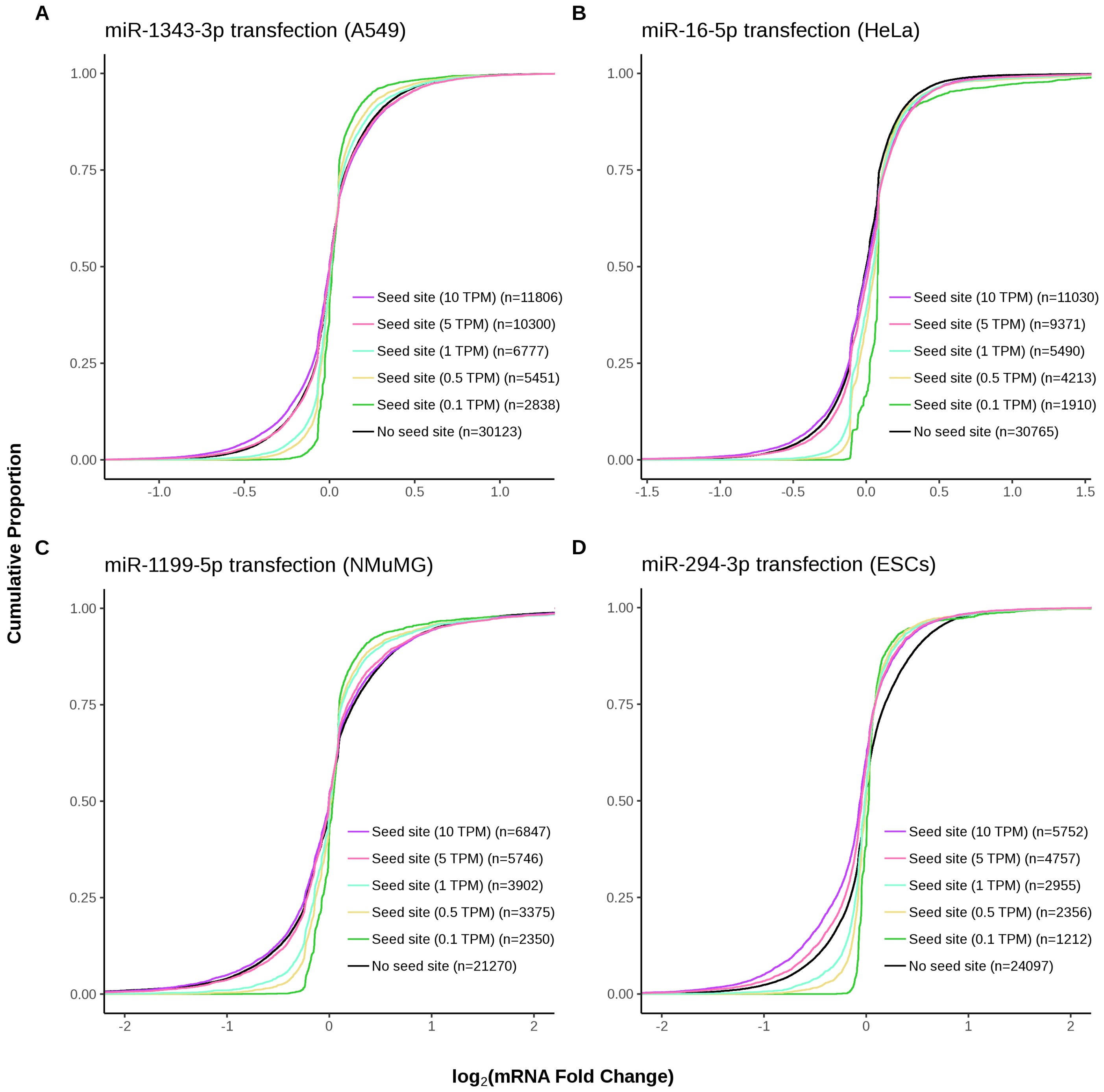

Supplementary Fig. S3b: The effect of expression filtering on removed protein-coding transcripts using multiple expression thresholds. Expression thresholds are implemented at TPM values of 10 (purple), 5 (pink), 1 (light blue), 0.5 (golden), and 0.1 (green). Otherwise as in Supplementary Figure S2.

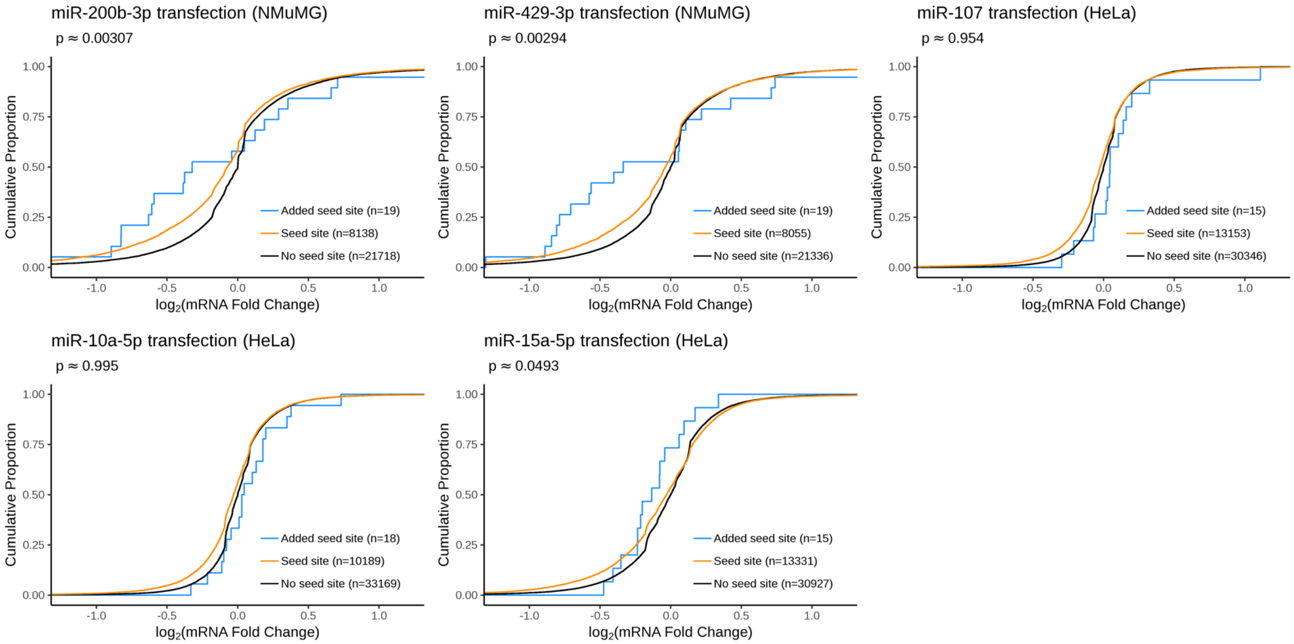

Supplementary Fig. S4 – As in Figure 2, though with additional number of datasets analysed.

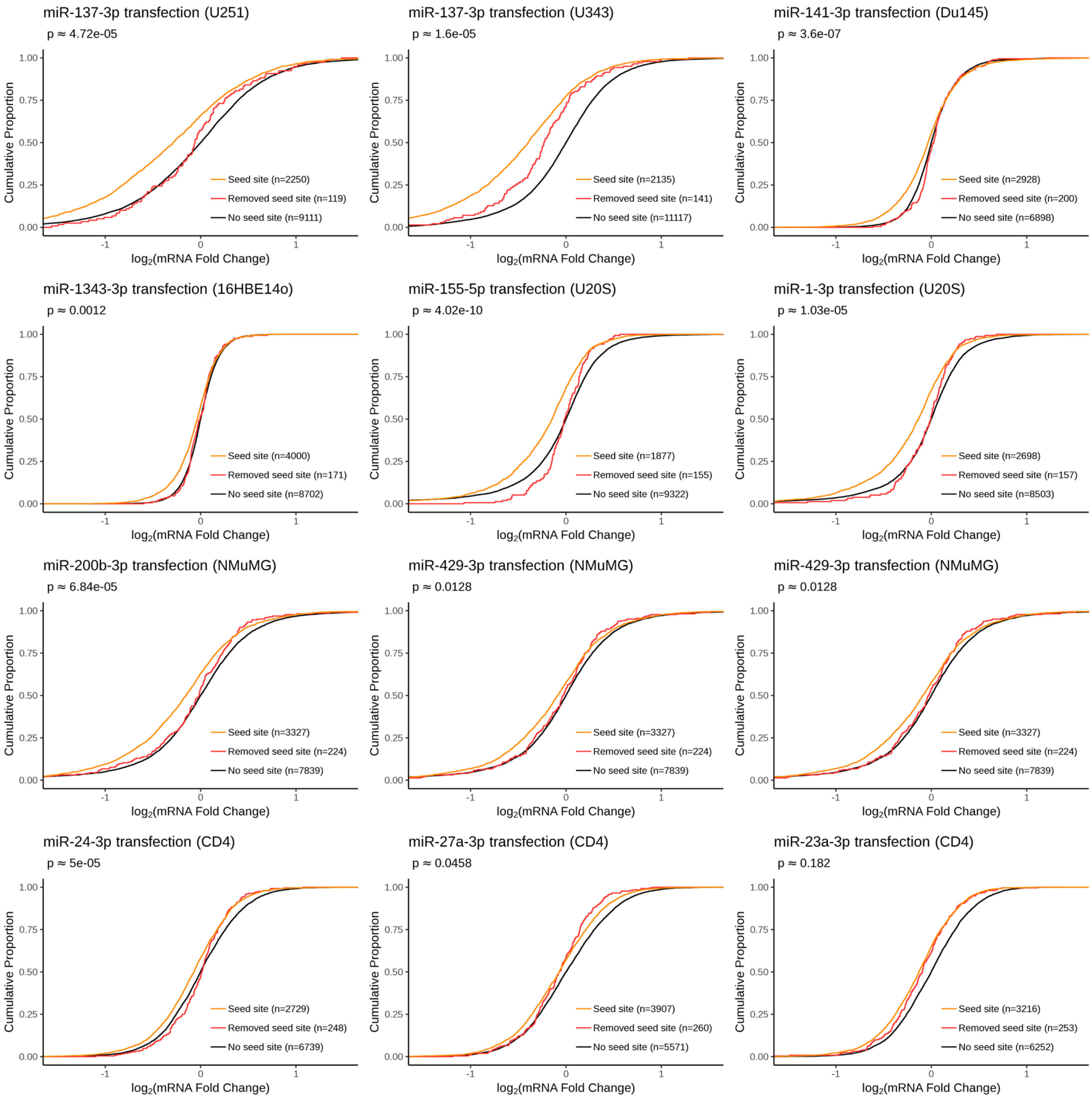

miR-137-3p transfection (U251)
p ≈ 4.72e-05
Seed site (n=2250)
Removed seed site (n=119)
No seed site (n=9111)
miR-137-3p transfection (U343)
p ≈ 1.6e-05
Seed site (n=2135)
Removed seed site (n=141)
No seed site (n=11117)
miR-141-3p transfection (Du145)
p ≈ 3.6e-07
Seed site (n=2928)
Removed seed site (n=200)
No seed site (n=6898)
miR-1343-3p transfection (16HBE14o)
p ≈ 0.0012
Seed site (n=4000)
Removed seed site (n=171)
No seed site (n=8702)
miR-155-5p transfection (U20S)
p ≈ 4.02e-10
Seed site (n=1877)
Removed seed site (n=155)
No seed site (n=9322)
miR-1-3p transfection (U20S)
p ≈ 1.03e-05
Seed site (n=2698)
Removed seed site (n=157)
No seed site (n=8503)
miR-200b-3p transfection (NMuMG)
p ≈ 6.84e-05
Seed site (n=3327)
Removed seed site (n=224)
No seed site (n=7839)
miR-429-3p transfection (NMuMG)
p ≈ 0.0128
Seed site (n=3327)
Removed seed site (n=224)
No seed site (n=7839)
miR-429-3p transfection (NMuMG)
p ≈ 0.0128
Seed site (n=3327)
Removed seed site (n=224)
No seed site (n=7839)
miR-24-3p transfection (CD4)
p ≈ 5e-05
Seed site (n=2729)
Removed seed site (n=248)
No seed site (n=6739)
miR-27a-3p transfection (CD4)
p ≈ 0.0458
Seed site (n=3907)
Removed seed site (n=260)
No seed site (n=5571)
miR-23a-3p transfection (CD4)
p ≈ 0.182
Seed site (n=3216)
Removed seed site (n=253)
No seed site (n=6252)
Cumulative Proportion
0.00
0.25
0.50
0.75
1.00
-1
0
1
log2(mRNA Fold Change)

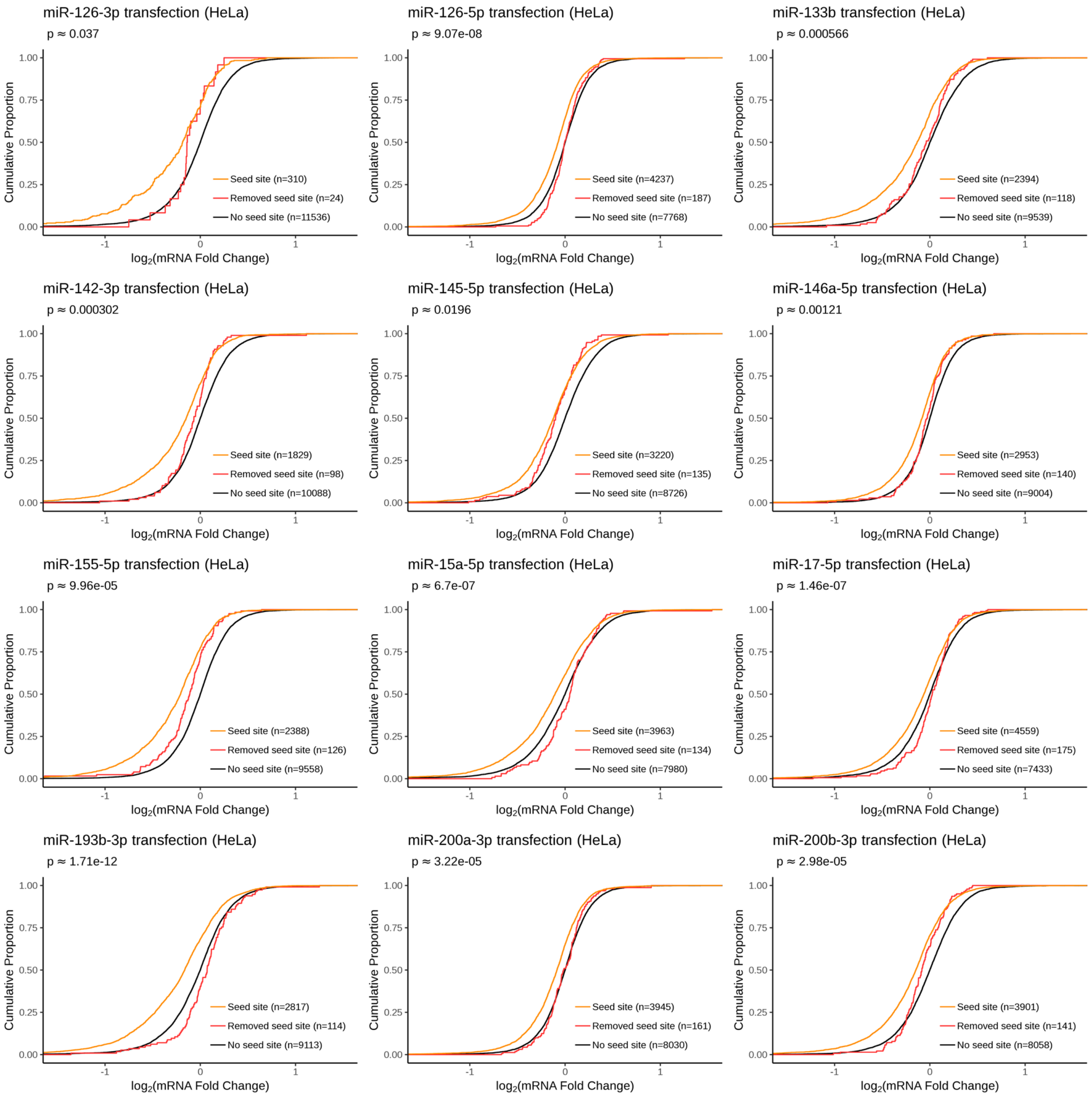
miR-126-3p transfection (HeLa)
p ≈ 0.037
Seed site (n=310)
Removed seed site (n=24)
No seed site (n=11536)
miR-126-5p transfection (HeLa)
p ≈ 9.07e-08
Seed site (n=4237)
Removed seed site (n=187)
No seed site (n=7768)
miR-133b transfection (HeLa)
p ≈ 0.000566
Seed site (n=2394)
Removed seed site (n=118)
No seed site (n=9539)
miR-142-3p transfection (HeLa)
p ≈ 0.000302
Seed site (n=1829)
Removed seed site (n=98)
No seed site (n=10088)
miR-145-5p transfection (HeLa)
p ≈ 0.0196
Seed site (n=3220)
Removed seed site (n=135)
No seed site (n=8726)
miR-146a-5p transfection (HeLa)
p ≈ 0.00121
Seed site (n=2953)
Removed seed site (n=140)
No seed site (n=9004)
miR-155-5p transfection (HeLa)
p ≈ 9.96e-05
Seed site (n=2388)
Removed seed site (n=126)
No seed site (n=9558)
miR-15a-5p transfection (HeLa)
p ≈ 6.7e-07
Seed site (n=3963)
Removed seed site (n=134)
No seed site (n=7980)
miR-17-5p transfection (HeLa)
p ≈ 1.46e-07
Seed site (n=4559)
Removed seed site (n=175)
No seed site (n=7433)
miR-193b-3p transfection (HeLa)
p ≈ 1.71e-12
Seed site (n=2817)
Removed seed site (n=114)
No seed site (n=9113)
miR-200a-3p transfection (HeLa)
p ≈ 3.22e-05
Seed site (n=3945)
Removed seed site (n=161)
No seed site (n=8030)
miR-200b-3p transfection (HeLa)
p ≈ 2.98e-05
Seed site (n=3901)
Removed seed site (n=141)
No seed site (n=8058)
Cumulative Proportion
0.00
0.25
0.50
0.75
1.00
-1
0
1
log2(mRNA Fold Change)

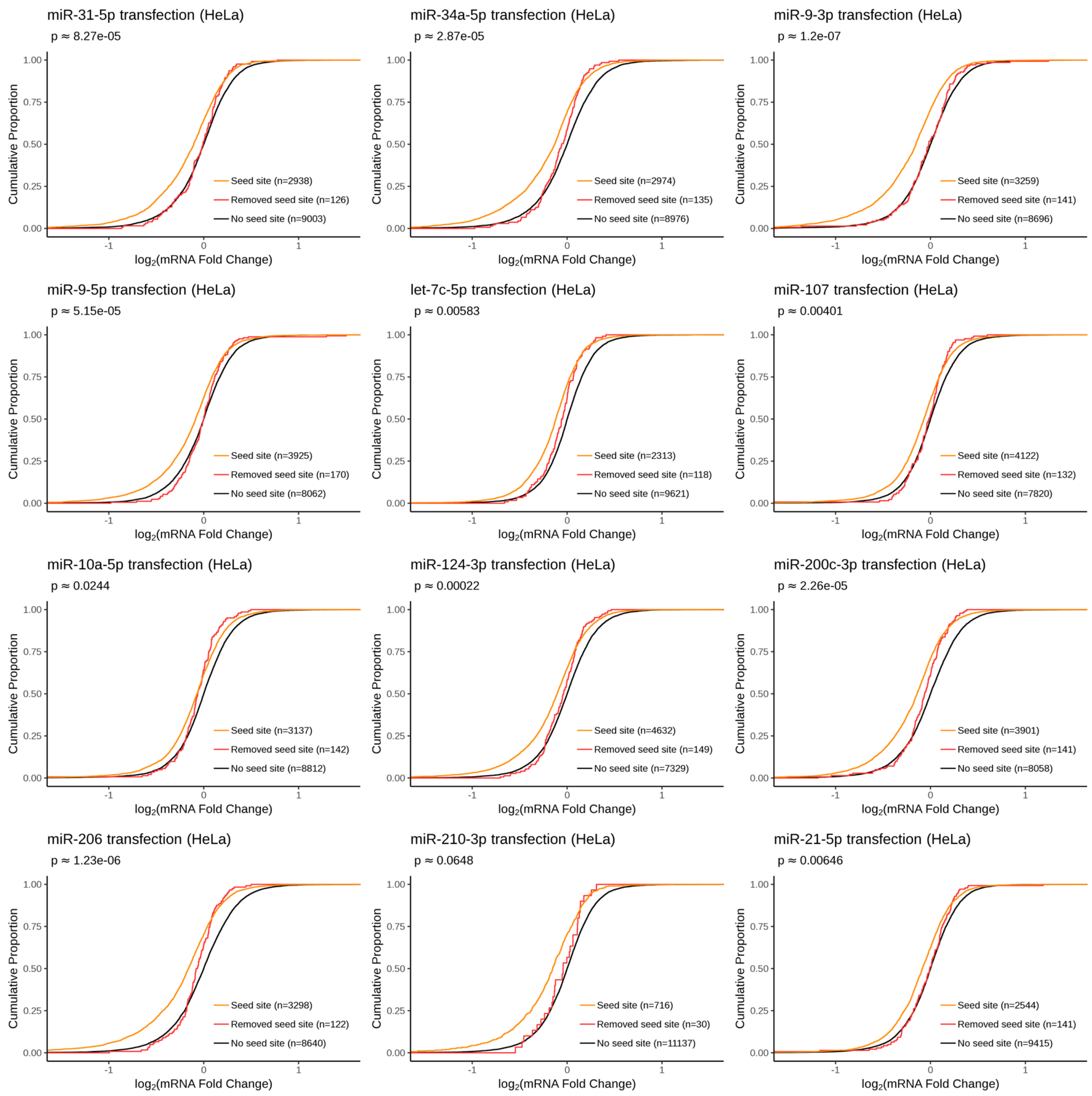

Supplementary Fig. S5 - As in Figure 3, though with additional datasets analysed.

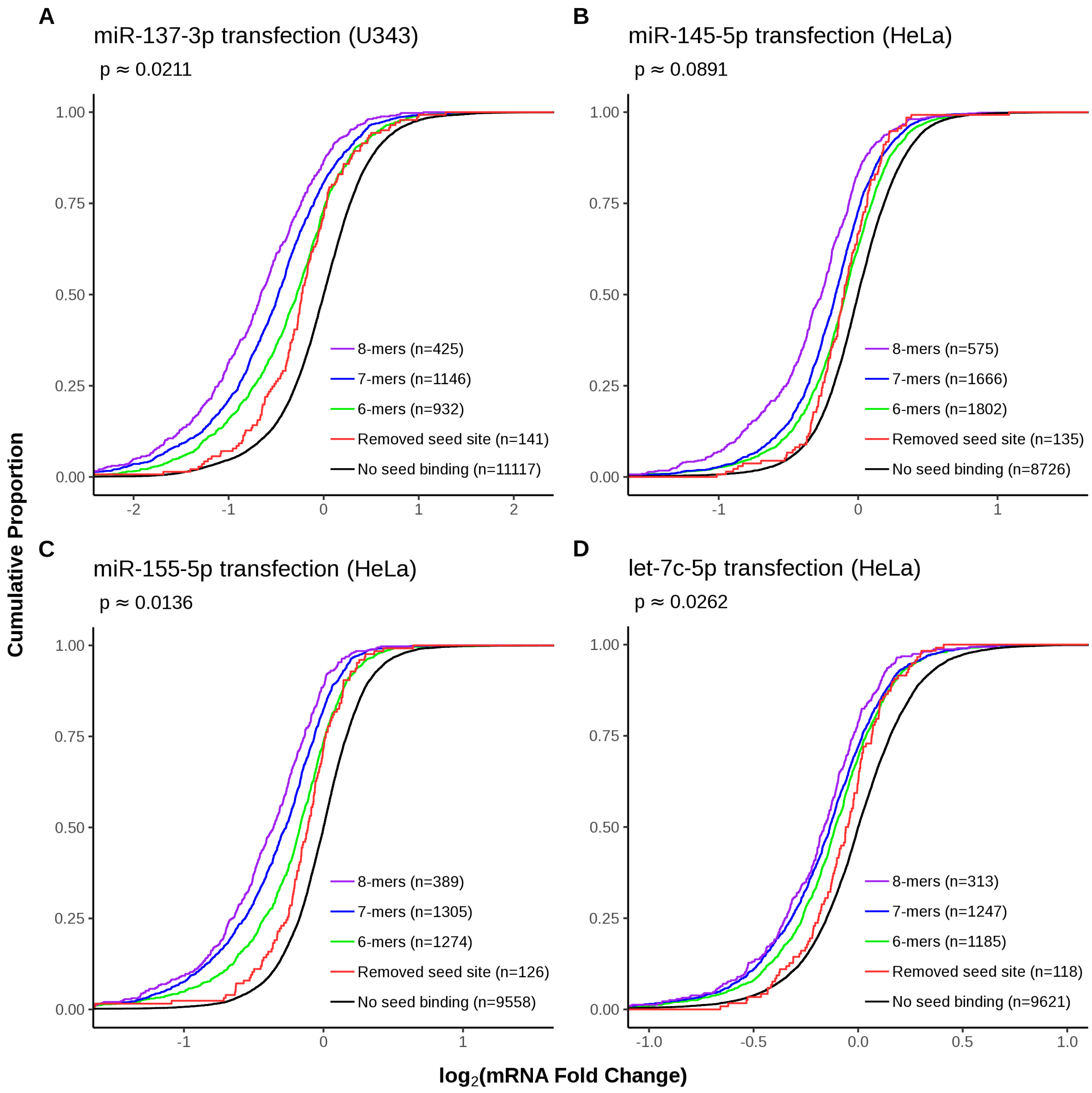

Supplementary Fig. S6: In experiments in which removed predicted target transcripts exhibit evidence of low-level repression, repression is less than that observed by transcripts targeted by marginally effective 6-mer seed sequences. As in Figure 3, with predicted target transcripts divided by miRNA target site type into 6-mer (green), 7-mer (blue) and 8-mer (purple) subsets. Approximate P-values were computed using one-sided, two-sample, Kolmogorov-Smirnov tests between discarded miRNA target and 6-mer target fold change distributions.

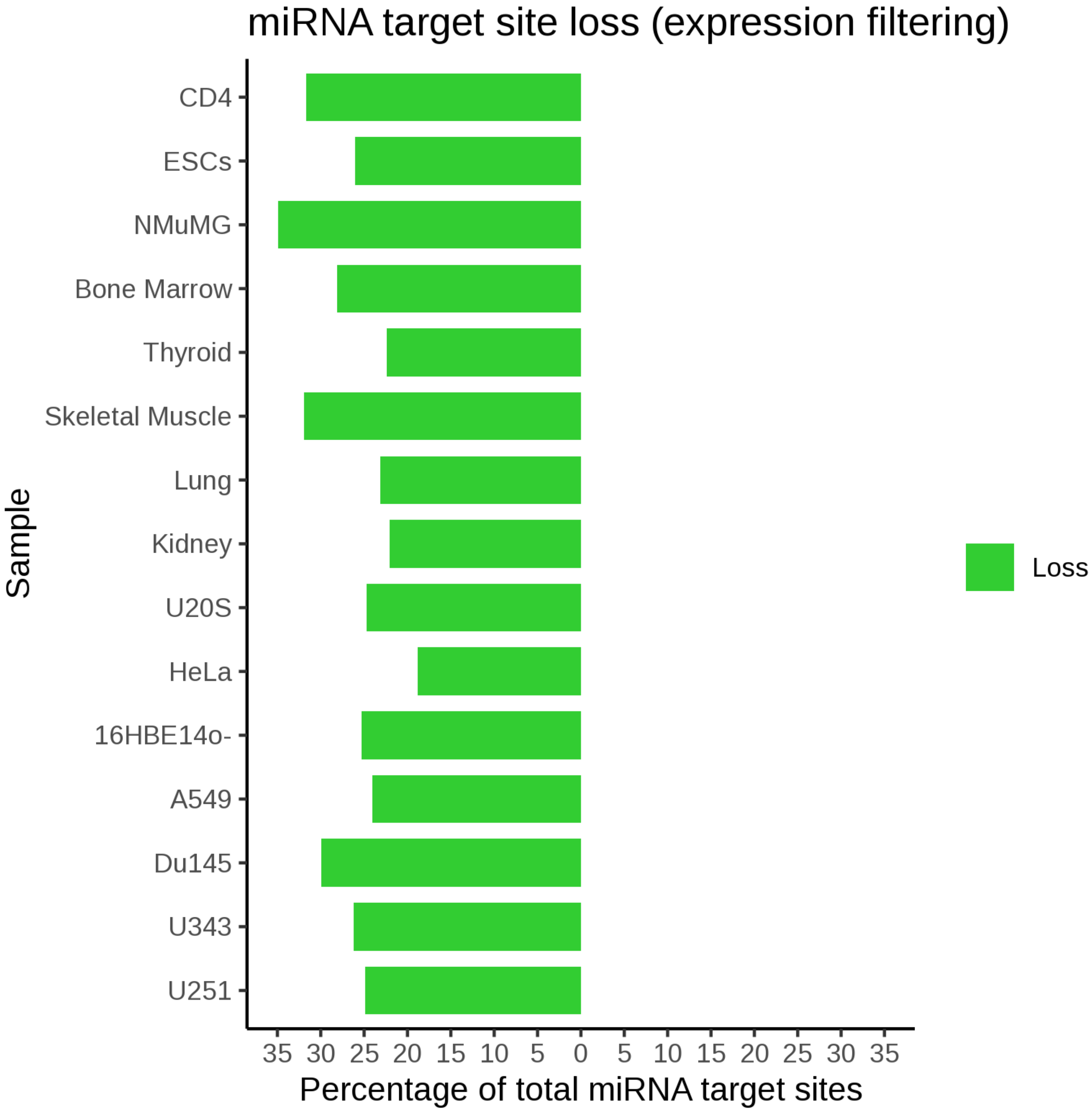

Supplementary Fig. S7: The percentage of total miRNA targets removed through expression filtering at a threshold of 0.1 TPM in a set of different cell lines and tissue types for human and mouse species.

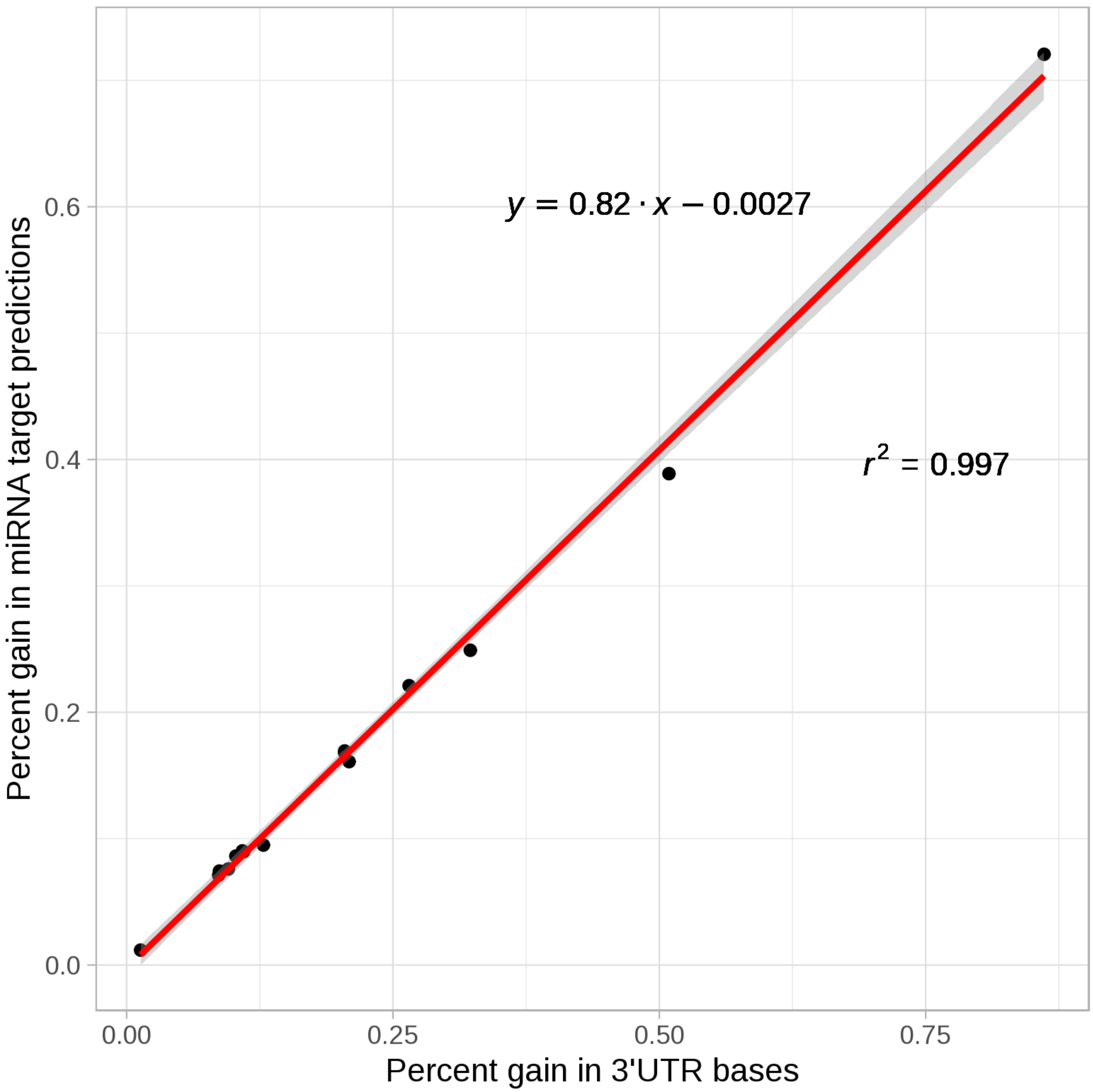

Supplementary Fig. S8a: A scatter plot of the percentage gain in total miRNA target site predictions vs. percentage gain in 3'UTR bases for a number of cell lines and tissue datasets analysed (black dots). A linear regression model was fitted using the 'lm' function of the R stats package (red) with a 95% confidence interval (grey). R-squared is derived from the Pearson correlation coefficient.

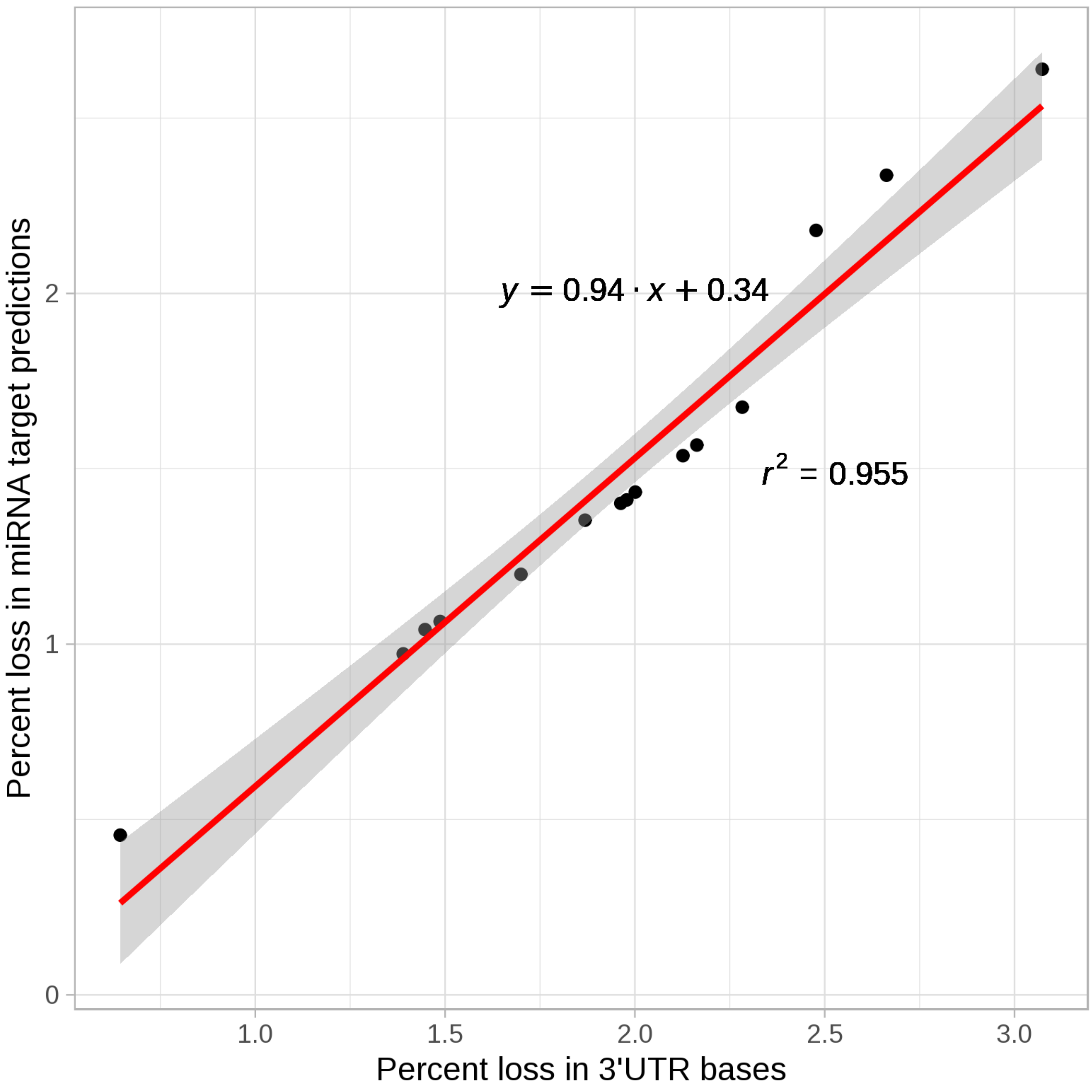

Supplementary Fig. S8b: A scatter plot of the percentage loss in total miRNA target predictions *vs.* percentage loss in total 3'UTR bases. Otherwise as in Supplementary Figure S8a.

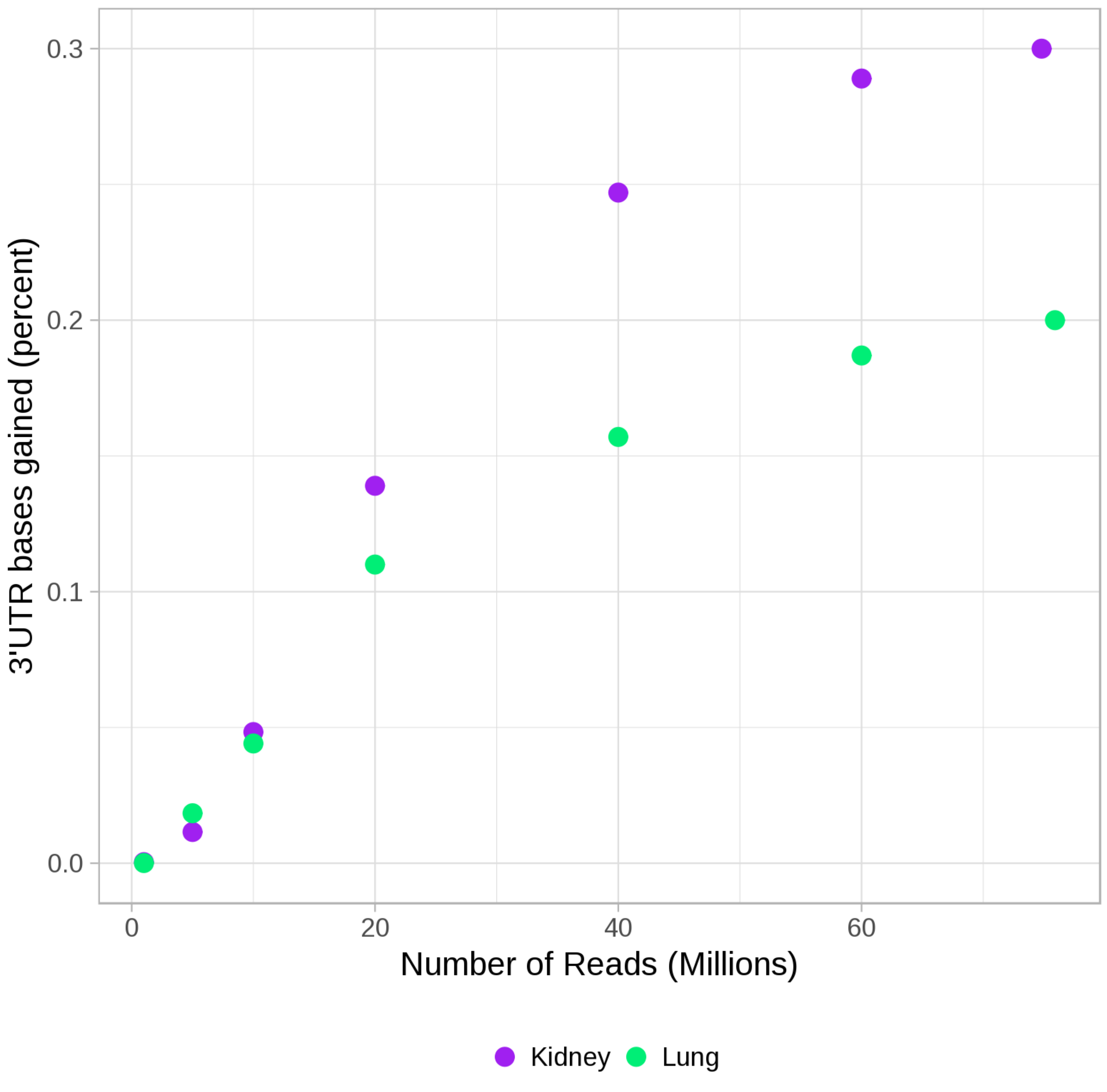

Supplementary Fig. S9a: The relationship between the number of reads sequenced and the extent of 3'UTR elongation observed when using FilTar for human kidney (purple) and lung (green) datasets. Variable read counts generated by randomly sampling reads from the total.

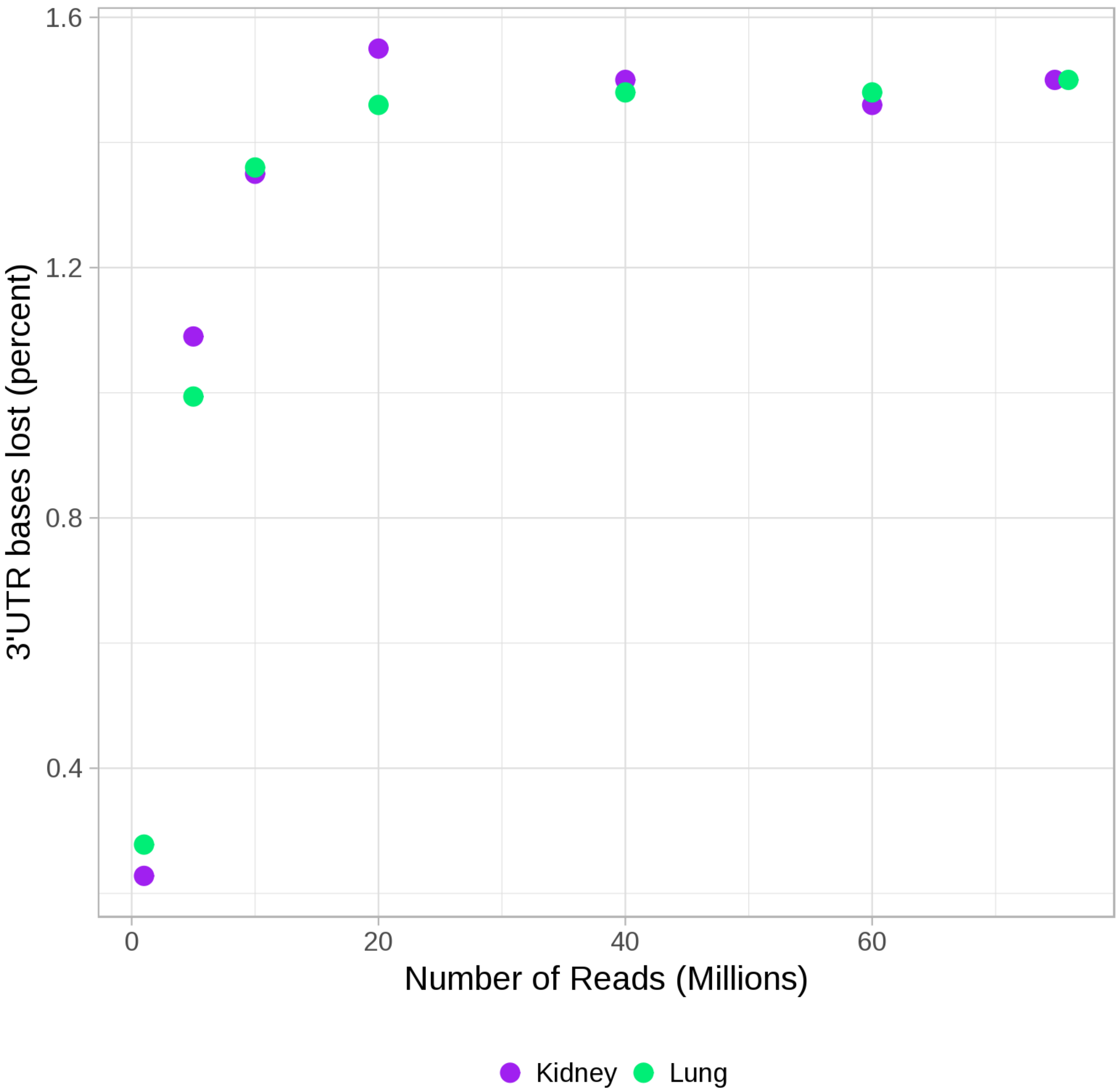

Supplementary Fig. S9b: The relationship between the number of reads sequenced and the extent of 3'UTR truncation observed when using FilTar within a given sample. Otherwise as in Supplementary Figure S9a.

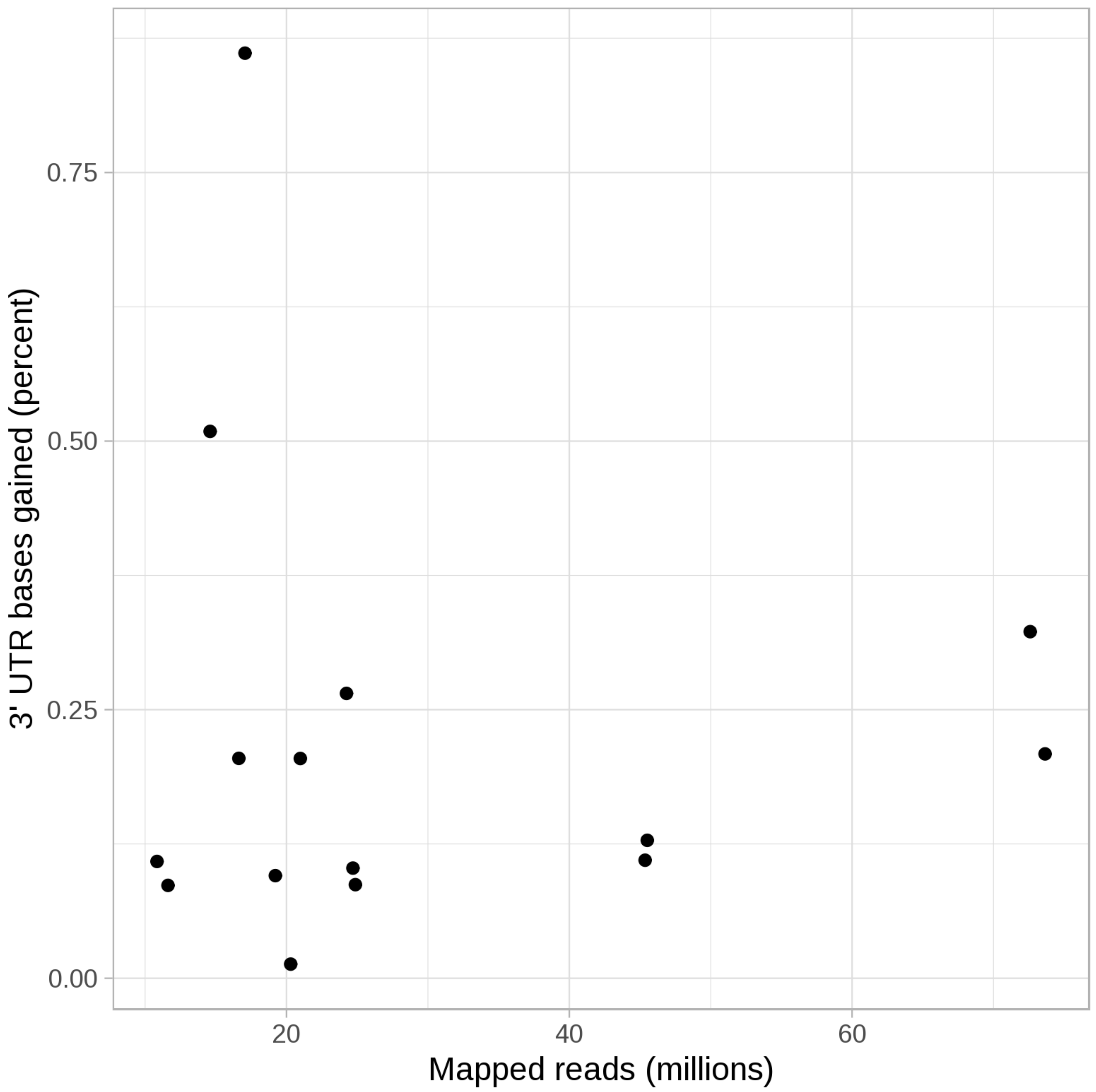

Supplementary Fig. S10a: The relationship between the number of mapped reads and the extent of 3'UTR elongation observed when using FilTar. Each point represents a different dataset analysed using FilTar. Supplementary Table S1 contains metadata for all datasets analysed.

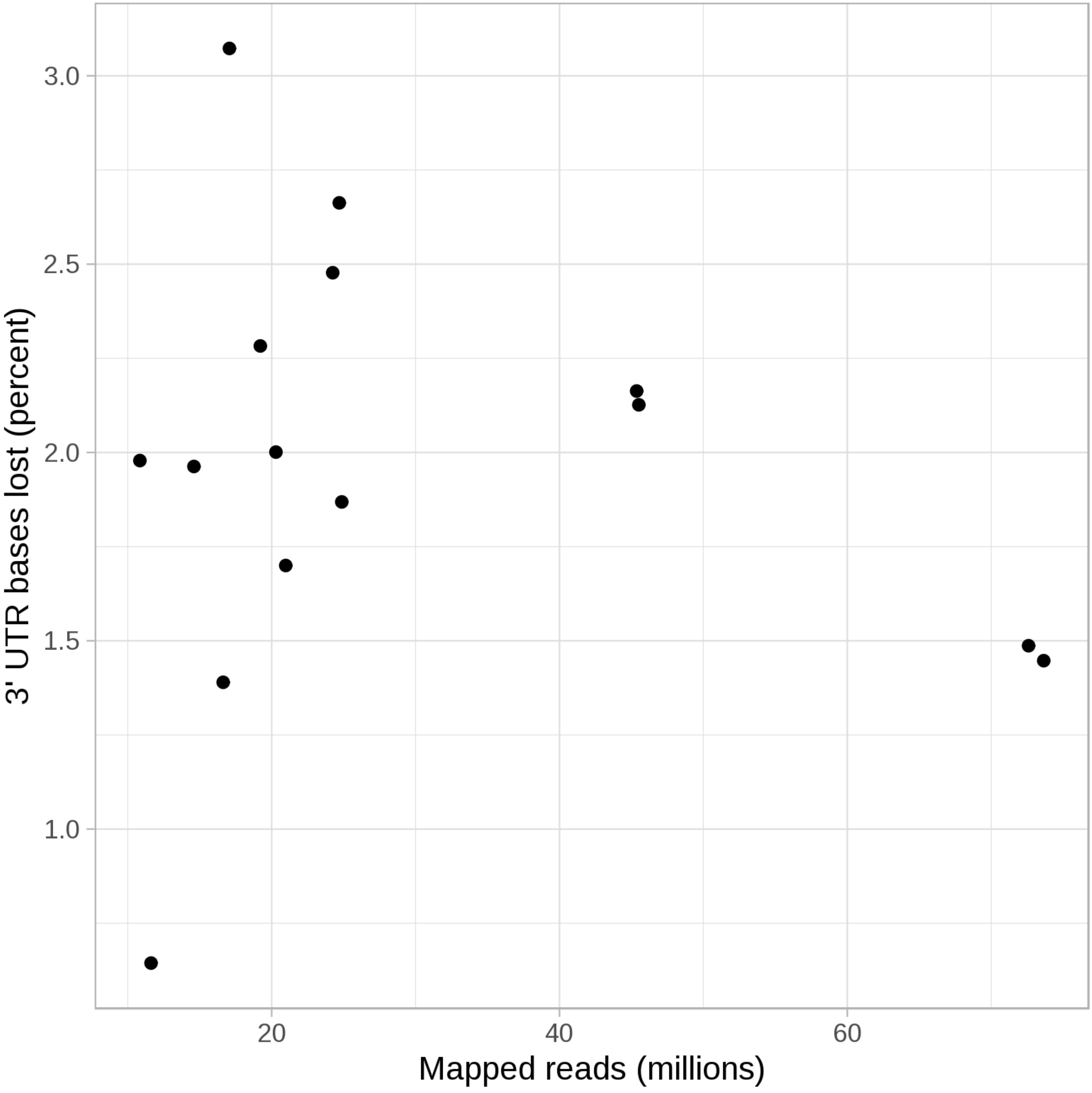

Supplementary Fig. S10b: The relationship between the number of mapped reads and the extent of 3'UTR truncation observed when using FilTar. Otherwise as in Supplementary Figure S10a.

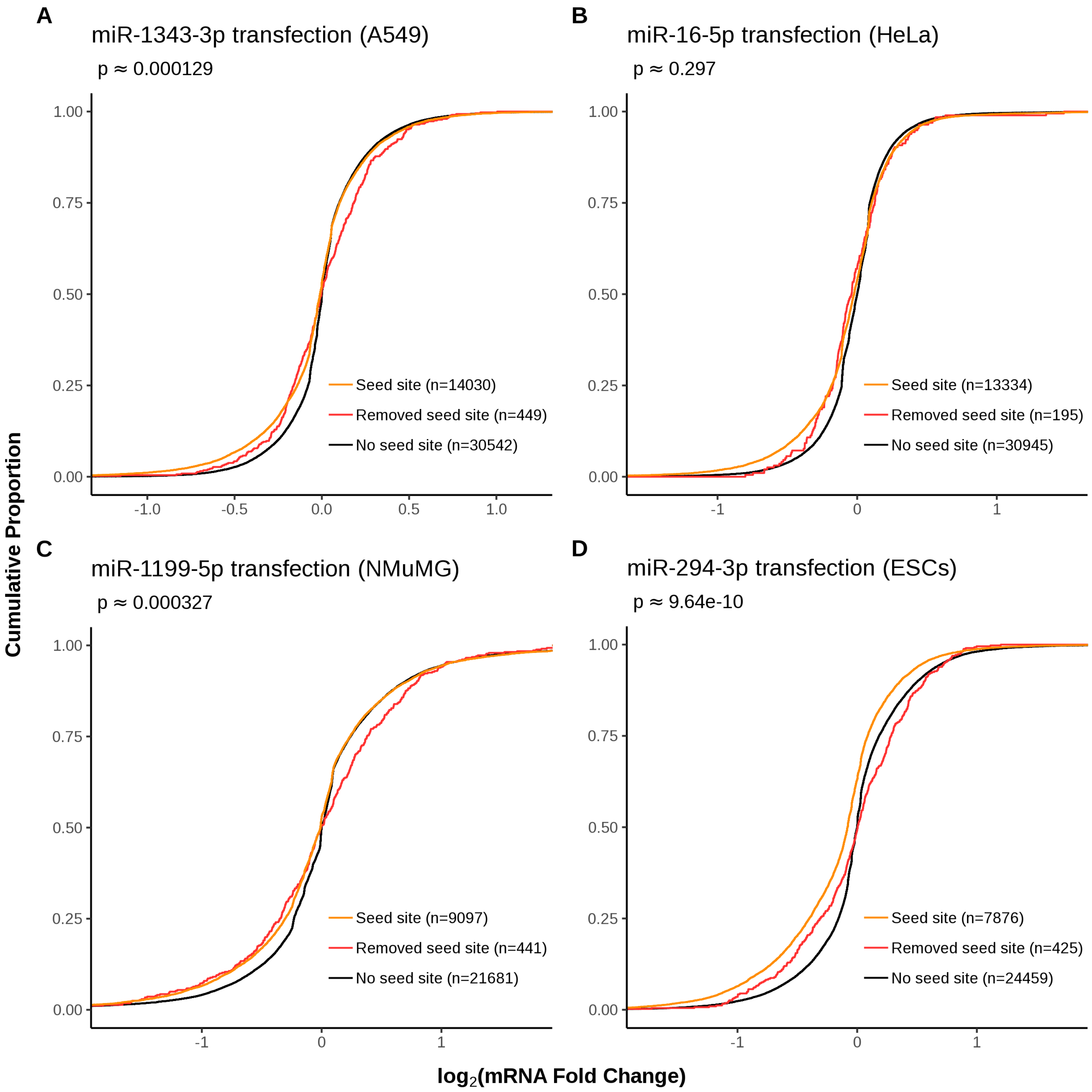

Supplementary Fig. S11: As in Figure 3, with the exception that no expression threshold has been implemented to filter data points contained within the removed seed site distribution.

Supplementary Tables

Supplementary Table S1: A summary of all datasets used in the analyses reported in this study

Species	BioProject Accession	Source/Study	Sample	Run Accessions
Homo sapiens	PRJNA231155	Tamim *et al.* 2014	U251	SRR1047622,SRR1047623,SRR1047624,SRR1047625
			U343	SRR1047630,SRR1047631,SRR1047632,SRR1047633
	PRJNA292016	Liu *et al.* 2017	Du145	SRR2146408,SRR2146409,SRR2146410,SRR2146411
	PRJNA304643	Stolzenburg *et al.* 2016	A549	SRR2968576,SRR2968577,SRR2968578,SRR2968579<br>SRR2968580,SRR2968581,SRR2968582,SRR2968583
			16HBE14o-	SRR2968584,SRR2968586,SRR2968588,SRR2968590<br>SRR2968592,SRR2968594,SRR2968596,SRR2968598
	PRJNA512378	Liu *et al.* 2019	HeLa	SRR8382192,SRR8382193,SRR8382194,SRR8382195<br>SRR8382196,SRR8382197,SRR8382198,SRR8382199<br>SRR8382200,SRR8382201,SRR8382202,SRR8382203<br>SRR8382204,SRR8382205,SRR8382206,SRR8382207<br>SRR8382208,SRR8382209,SRR8382210,SRR8382211<br>SRR8382212,SRR8382213,SRR8382214,SRR8382215<br>SRR8382216,SRR8382217,SRR8382218,SRR8382219<br>SRR8382220,SRR8382221,SRR8382222,SRR8382223<br>SRR8382224,SRR8382225,SRR8382226,SRR8382227<br>SRR8382228,SRR8382229,SRR8382230,SRR8382231<br>SRR8382232,SRR8382233,SRR8382234,SRR8382235<br>SRR8382236,SRR8382237,SRR8382238,SRR8382239<br>SRR8382240,SRR8382241,SRR8382242,SRR8382243
	PRJNA223608	Guo *et al.* 2014	U20S	SRR1598955,SRR1598970,SRR1598976,SRR1598977<br>SRR1598972,SRR1598973
	PRJEB2445	Illumina BodyMap2 transcriptome	Kidney	ERR030885,ERR030893
			Lung	ERR030879,ERR030896
	PRJEB6971	Science for Life Laboratory, Stockholm	Skeletal Muscle	ERR579142,ERR579143
			Thyroid	ERR315358,ERR315422
			Bone Marrow	ERR315404,ERR315406
Mus Musculus	PRJNA340017	Diepenbruck *et al.* 2017	NMuMG	SRR4054984,SRR4054985,SRR4054992,SRR4054995<br>SRR4054996,SRR4054999,SRR4055002,SRR4055005
	PRJNA309441	Pua *et al.* 2016	CD4+	SRR3112249,SRR3112250,SRR3112251,SRR3112252<br>SRR3112245,SRR3112246,SRR3112247,SRR3112248<br>SRR3112237,SRR3112238,SRR3112239,SRR3112240<br>SRR3112241,SRR3112242,SRR3112243,SRR3112244
	PRJNA270999	Cao *et al.* 2015	ESCs	SRR1734389,SRR1734391,SRR1734393,SRR1734395

Supplementary Table S2 - A table of summary and quality control statistics for all sequencing runs used in this analysis. Statistics are given for the percentage of sequenced reads pseudo-aligned to the transcriptome using kallisto. The estimated mean average fragment length is given for sequencing runs in which cDNA libraries are sequenced using paired-end sequencing protocols. For single-end sequencing protocols, fragment length statistics cannot be inferred. The percentage of reads aligned to the relevant genome using HISAT2 (for the purposes of 3'UTR reannotation) is also given. Other QC statistics such as the percentage of reads trimmed, the percentage of reads which are duplicates, and the mean percentage GC content of reads is also reported. In the final two columns, the length of reads, and the number of reads sequenced for each sequencing run is given.

Accession	Fragment Length	% Kallisto aligned	% HISAT2 aligned	% trimmed	% dups	% GC	Length	Total Reads (M)
ERR030888	NA	83.20	96.60	4.1	44.4	47	73	74.6
ERR030893	NA	82.20	95.60	4.5	24.4	45	73	77.6
ERR030896	NA	87.90	97.00	4.4	47.3	46	74	78.7
SRR1047622	NA	50.70	93.40	0.9	51.5	46	36	49.6
SRR1047623	NA	57.00	92.00	1	48.4	45	36	48.6
SRR1047624	NA	39.50	92.80	0.8	45	46	36	40.3
SRR1047625	NA	35.90	94.20	0.9	53.6	48	36	45
SRR1047630	NA	74.00	72.30	0.6	59	51	36	67.6
SRR1047631	NA	72.30	71.90	0.4	59.6	51	36	58.2
SRR1047632	NA	72.00	72.70	0.6	60.9	52	36	63.4
SRR1047633	NA	72.20	72.30	0.7	59.1	51	36	67.1
SRR1598955	NA	54.30	89.20	10	44.4	48	36	19.5
SRR1598970	NA	37.90	87.90	17.9	77.8	49	36	26.4
SRR1598972	NA	53.70	89.70	10.9	47.9	48	36	20.7
SRR1598973	NA	35.30	38.90	23.2	79.2	50	49	74.8
SRR1598976	NA	52.20	91.00	9.9	59.2	48	36	19.9
SRR1598977	NA	34.60	87.60	16.9	79.7	49	36	26.1
SRR8382192	NA	41.50	94.00	0.6	36	47	50	25.5
SRR8382193	NA	42.40	94.90	0.6	40.8	46	50	31.8
SRR8382194	NA	41.50	93.70	0.8	33.9	47	50	23.9
SRR8382195	NA	43.30	94.00	1.4	40.5	47	50	25.1
SRR8382196	NA	44.00	94.00	0.5	34.4	46	50	22.9
SRR8382197	NA	44.20	94.20	5	55.5	47	50	22.9
SRR8382198	NA	41.00	93.90	0.6	33.1	46	50	25.6
SRR8382199	NA	42.60	94.30	0.8	36.6	47	50	26.7
SRR8382200	NA	38.40	93.10	1.8	41.3	46	50	27.7

SRR8382201	NA	39.40	93.50	1	43	46	50	30.8
SRR8382202	NA	40.50	93.50	0.5	35	46	50	25.5
SRR8382203	NA	38.50	93.80	0.9	39.4	46	50	27.9
SRR8382204	NA	42.40	94.00	0.4	33.8	46	50	23.4
SRR8382205	NA	41.40	92.90	1	49.6	48	50	24.4
SRR8382206	NA	41.90	93.90	0.4	34.4	46	50	24.4
SRR8382207	NA	42.30	94.30	0.7	39	46	50	25
SRR8382208	NA	45.00	94.20	0.4	35	47	50	22.6
SRR8382209	NA	44.60	94.20	0.7	38	48	50	24.1
SRR8382210	NA	39.20	93.60	0.6	34.1	45	50	26.1
SRR8382211	NA	39.00	93.60	1.2	40.9	46	50	28.6
SRR8382212	NA	38.70	93.70	0.5	34	46	50	27.5
SRR8382213	NA	43.30	94.90	0.4	37.2	46	50	27.4
SRR8382214	NA	31.70	92.40	0.6	32.8	46	50	35
SRR8382215	NA	41.80	94.40	1.2	37.3	46	50	25.9
SRR8382216	NA	33.90	93.30	1.2	32.2	45	50	30.7
SRR8382217	NA	40.10	94.60	0.7	36.2	45	50	29.1
SRR8382218	NA	34.40	93.20	0.5	31.7	45	50	30.6
SRR8382219	NA	45.70	94.60	0.8	37.8	47	50	23.9
SRR8382220	NA	31.00	92.70	0.7	36.3	45	50	36.2
SRR8382221	NA	35.30	93.70	0.5	37.6	45	50	33
SRR8382222	NA	39.60	93.70	0.4	34.2	46	50	25
SRR8382223	NA	39.80	93.50	2.5	41	47	50	27.4
SRR8382224	NA	41.40	94.00	0.5	33.8	46	50	26.1
SRR8382225	NA	44.70	95.00	1	37.8	46	50	25.1
SRR8382226	NA	43.80	94.50	0.8	36.4	47	50	23
SRR8382227	NA	42.10	94.50	0.4	34.4	46	50	26.1
SRR8382228	NA	38.70	94.00	0.5	32.3	46	50	29.1
SRR8382229	NA	43.40	94.10	1.6	48.7	46	50	28.3
SRR8382230	NA	35.70	93.50	0.6	31.2	46	50	28.8
SRR8382231	NA	42.70	94.90	0.9	36	46	50	25.8
SRR8382232	NA	36.30	92.80	1.6	34	46	50	29.6
SRR8382233	NA	39.40	94.10	1.1	40.6	45	50	27.7
SRR8382234	NA	38.10	93.10	1.6	34.7	46	50	27.4
SRR8382235	NA	38.00	93.50	1	41	45	50	29.9
SRR8382236	NA	40.30	93.90	0.6	33.3	46	50	25.3
SRR8382237	NA	42.00	94.80	0.6	37.5	45	50	26.5
SRR8382238	NA	39.40	94.00	0.5	35.6	45	50	25.4
SRR8382239	NA	41.60	93.70	1.2	44	46	50	26.1
SRR8382240	NA	40.20	93.60	0.5	37	46	50	26.4
SRR8382241	NA	39.40	93.70	1.1	41.3	47	50	27
SRR8382242	NA	40.80	93.80	0.5	35	46	50	25
SRR8382243	NA	39.00	93.30	1.4	43.7	47	50	28.2
SRR3112237	NA	33.90	67.30	1.7	39.1	45	51	36.1
SRR3112238	NA	29.70	83.40	1.1	25.5	41	51	17
SRR3112239	NA	32.30	90.70	1.1	35.3	43	51	36.5

SRR3112240	NA	36.40	90.40	1	22.9	42	51	37.4
SRR3112241	NA	29.40	74.80	1.3	36.4	39	51	26.6
SRR3112242	NA	28.50	88.40	1.1	40.3	40	51	46.8
SRR3112243	NA	32.30	79.70	1.4	37.7	45	51	37.4
SRR3112244	NA	32.60	77.50	1.3	30.5	44	51	31.9
SRR3112245	NA	35.20	80.30	1.2	17.2	43	51	8.9
SRR3112246	NA	40.80	90.50	1	17.4	42	51	5.6
SRR3112247	NA	36.80	89.30	1.1	53.1	43	51	37.7
SRR3112248	NA	32.50	85.80	1.2	29	40	51	22.3
SRR3112249	NA	31.20	85.20	1	23.7	40	51	0.2
SRR3112250	NA	32.00	91.60	1	44.3	40	51	37.4
SRR3112251	NA	33.70	84.60	1.1	55.7	40	51	40.3
SRR3112252	NA	29.00	87.50	1.1	39.6	39	51	34.6
SRR4054984	NA	86.20	94.20	0.7	57.7	48	51	37.4
SRR4054985	NA	87.90	96.20	0.4	48.6	49	51	13.8
SRR4054992	NA	88.70	95.50	0.4	53	49	51	16.5
SRR4054995	NA	88.90	94.70	0.9	61.7	49	51	42.6
SRR4054996	NA	86.90	95.20	0.8	47.4	48	51	13.7
SRR4054999	NA	87.60	95.80	0.4	47.7	49	51	13.5
SRR4055002	NA	87.60	95.80	0.4	47.9	49	51	13.6
SRR4055005	NA	87.80	95.80	0.4	46.5	49	51	11.1
ERR030879	173.1	89.00	97.20	4.75	54.9	46	49.5	73
ERR030880	255.2	82.80	96.70	3.55	56.2	47	49.5	71.9
ERR030885	212.2	81.70	95.90	4.05	54.4	45	49.5	74
ERR315358	322.8	86.30	96.30	3.55	38.35	45	99	15.2
ERR315404	204.7	80.80	98.20	4	39.9	48	97.5	16.8
ERR315406	205.2	80.80	98.20	4.05	40.4	48	97.5	17
ERR315422	323.3	86.30	96.30	3.4	38.65	45	99	15.1
ERR579142	120.8	90.90	96.70	47.75	53.5	50	89.5	16
ERR579143	125.6	91.00	95.70	40.95	45	49	89.5	8.1
SRR2146408	179.6	35.60	51.40	3.05	66.5	44.5	75	33.2
SRR2146409	176.8	41.40	57.90	2.7	63.6	45	75	36.9
SRR2146410	171.7	41.20	60.00	2.6	63.9	44.5	75	36.9
SRR2146411	171.6	47.80	70.30	2.8	59.85	45	75	34.7
SRR2968576	179.8	87.60	98.30	1.55	46.4	49	48	24
SRR2968577	173.4	88.10	98.30	1.85	44.7	49	48	20
SRR2968578	170.8	88.20	98.30	1.7	42.65	49	48	17.1
SRR2968579	182.4	77.90	98.30	1.65	50.2	50	48	22.2
SRR2968580	180.8	86.00	98.40	1.3	50.65	49	48	31
SRR2968581	178.9	88.30	98.30	1.4	49	49	48	32.7
SRR2968582	184.8	88.70	98.30	1.4	44.05	49	48	21.4
SRR2968583	184.9	87.90	98.20	2.3	40.4	48	48	17.1
SRR2968584	225.0	85.70	90.00	8.55	29.25	49	47.5	12.7
SRR2968586	213.3	86.10	90.10	8.95	29.15	49	47.5	11.9
SRR2968588	206.5	86.30	90.30	7.75	27.15	49	47.5	9.9
SRR2968590	195.7	85.20	90.00	8.6	30.85	49	47.5	13.7

SRR2968592	202.5	82.80	90.10	8.75	29.25	50	47.5	11.7
SRR2968594	200.9	85.30	90.00	8.55	30.55	49	47.5	15.2
SRR2968596	209.6	85.30	89.80	9	29.15	49	47.5	14.1
SRR2968598	191.9	84.20	89.90	9.3	30.35	49	47.5	13.7
SRR1734389	182.4	88.10	98.40	3.1	46.3	49.5	98.5	17.5
SRR1734391	184.1	87.30	98.20	2.95	45.45	48	98.5	17.3
SRR1734393	188.6	88.90	98.00	2.85	48.1	49.5	98.5	15.7
SRR1734395	182.8	88.60	98.00	3.1	50.5	49	98.5	18.1

Supplementary Table S3: For the columns labelled ‘pre-existing 3’UTR annotation’ or similar: The number and percentage of protein-coding transcripts with a pre-existing 3’UTR annotation for each sample type after expression filtering at TPM > 0.1. For the columns labelled ‘Single exon 3’UTRs’ or similar: The number and percentage of protein-coding transcripts with a pre-existing annotated 3’UTR which does not span more than one exon, for each sample type after expression filtering at TPM > 0.1. The final column, labelled ‘Protein-coding transcripts used (%)’ gives the percentage of total protein-coding transcripts which are used as input for the reannotation step of the FilTar workflow. It is the product of the two other percentage columns given in this table.

**Species**	**Samples**	**Pre-existing 3’UTR annotation**	**Pre-existing 3’UTR annotation (%)**	**Single exon 3’UTRs**	**Single exon 3’UTRs (%)**	**Protein-coding transcripts used (%)**
Homo sapiens	U251	29472	73.5	27538	93.4	68.7
	U343	28810	70.2	26919	93.4	65.6
	Du145	30795	81.3	28739	93.3	75.8
	A549	35966	77.8	33595	93.4	72.7
	16HBE14o-	35727	76.2	33336	93.3	71.1
	HeLa	31379	72.5	29362	93.6	67.9
	U20S	29178	73.8	27209	93.3	68.8
	Kidney	34187	84.4	31991	93.6	78.9
	Lung	33800	84.4	31630	93.6	79.0
	Skeletal Muscle	26197	83.6	24509	93.6	78.2
	Thyroid	34479	81.6	32262	93.6	76.4
	Bone Marrow	32886	78.1	30675	93.3	72.9
Mus musculus	NMuMG	20622	77.9	19460	94.4	73.5
	CD4+	20985	83.1	19818	94.4	78.5
	ESCs	25854	81.2	24444	94.5	76.7

Supplementary Table S4: The total number of miRNA seed sites lost through expression filtering of transcripts at TPM > 0.1 or gained and lost through 3'UTR reannotation. Total miRNA seed sites for human: 52084138 and mouse: 28216437.

**Species**	**Samples**	**Seed sites gained (3'UTR reannotation)**	**Seed sites lost (3'UTR reannotation)**	**Seed sites lost (expression filtering)**
Homo sapiens	U251	49345	800764	12942294
	U343	46701	816545	13657488
	Du145	39571	872804	12508511
	A549	87549	624503	15578814
	16HBE14o-	47031	735041	13193677
	HeLa	38712	704948	9792951
	U20S	6146	746686	12879630
	Kidney	129715	554534	11476758
	Lung	83821	542432	12057289
	Skeletal muscle	37028	237223	16615464
	Thyroid	202504	730038	11682705
	Bone marrow	88212	506415	14632213
Mus musculus	NMuMG	62367	615046	9858668
	CD4+	203359	744867	7358255
	ESCs	24318	659420	8947356

Supplementary Table S5: Summary statistics of the effects of filtering protein-coding transcripts at an expression threshold of 0.1 TPM. Statistics are for the total number and proportion of bases and transcripts removed as a result of expression filtering.

**Species**	**Samples**	**Bases lost (Mb)**	**Bases lost (%)**	**3'UTRs removed**	**3' UTRs removed (%)**
Homo sapiens	U251	19.56	32.0	22653	42.0
	U343	21.07	34.4	21929	40.6
	Du145	24.06	39.3	25494	47.2
	A549	19.50	31.9	20783	38.5
	16HBE14o-	20.73	33.9	21221	39.3
	HeLa	15.09	24.6	18907	35.0
	U20S	19.96	32.6	22548	41.8
	Kidney	17.78	29.0	22476	41.6
	Lung	18.68	30.5	22647	42.0
	Skeletal muscle	25.86	42.2	28148	52.1
	Thyroid	17.84	29.1	21529	39.9
	Bone marrow	22.78	37.2	23040	42.7
Mus musculus	NMuMG	20.66	43.2	19592	47.7
	CD4+	18.61	38.9	19862	48.5
	ESCs	15.61	32.6	15505	37.9

Supplementary Table S6: FilTar 3'UTR reannotation summary statistics for cell line and tissue data used in this study. Statistics are the total number or proportion of bases or transcripts gained or lost through 3'UTR reannotation respectively. All comparisons are made against a reference of Ensembl annotated 3'UTR sequences associated exclusively with protein-coding mRNA transcripts.

Species	Samples	Bases gained (Mb)	Bases gained (%)	Bases lost (Mb)	Bases lost (%)	3' UTRs elongated	3' UTRs elongated (%)	3'UTRs truncated	3' UTRs truncated (%)
Homo sapiens	U251	0.08	0.1	1.30	2.1	352	0.7	5730	10.6
	U343	0.07	0.1	1.32	2.2	296	0.5	7395	13.7
	Du145	0.06	0.1	1.40	2.3	453	0.8	5342	9.9
	A549	0.13	0.2	1.04	1.7	281	0.5	6774	12.5
	16HBE14o-	0.07	0.1	1.21	2.0	213	0.4	6600	12.2
	HeLa	0.05	0.1	1.14	1.9	289	0.5	4087	7.6
	U20S	0.01	0.0	1.23	2.0	120	0.2	3614	6.7
	Kidney	0.20	0.3	0.91	1.5	708	1.3	5738	10.6
	Lung	0.13	0.2	0.89	1.4	538	1.0	5686	10.5
	Skeletal muscle	0.05	0.1	0.39	0.6	136	0.3	3018	5.6
	Thyroid	0.31	0.5	1.20	2.0	460	0.9	7356	13.6
	Bone marrow	0.13	0.2	0.85	1.4	292	0.5	5444	10.1
Mus musculus	NMuMG	0.13	0.3	1.18	2.5	454	1.1	6440	15.8
	CD4+	0.05	0.1	1.27	2.7	345	0.8	2447	6.0
	ESCs	0.41	0.9	1.46	3.1	493	1.2	7502	18.4

Supplementary Table S7: Summed statistics from Supplementary Tables S5 and S6 relating to total combined 3'UTR bases and 3'UTRs affected by expression filtering and 3'UTR truncation.

**Species**	**Samples**	**Bases lost (Mb)**	**Bases lost (%)**	**3'UTRs affected**	**3' UTRs affected (%)**
Homo sapiens	U251	20.86	34.1	28383	52.6
	U343	22.39	36.6	29324	54.3
	Du145	25.45	41.6	30836	57.1
	A549	20.54	33.5	27557	51.1
	16HBE14o-	21.94	35.8	27821	51.5
	HeLa	16.23	26.5	22994	42.6
	U20S	21.19	34.6	26162	48.5
	Kidney	18.69	30.5	28214	52.3
	Lung	19.57	32.0	28333	52.5
	Skeletal muscle	26.26	42.9	31166	57.7
	Thyroid	19.04	31.1	28885	53.5
	Bone marrow	23.63	38.6	28484	52.8
Mus musculus	NMuMG	21.84	45.7	25969	63.5
	CD4+	19.87	41.6	22309	54.5
	ESCs	17.07	35.7	23007	56.3
